# Integrated multi-omics analysis identifies candidate eRNA-associated signatures shared between osteoarthritis and type 2 diabetes

**DOI:** 10.3389/fgene.2026.1875546

**Published:** 2026-07-17

**Authors:** Danni Huang, Junying Wu, Jinhua Chen, Qian Wu, Shengnan Wang

**Affiliations:** 1 Division of Orthopaedics and Traumatology, Department of Orthopaedics, Nanfang Hospital, Southern Medical University, Guangzhou, Guangdong Province, China; 2 Department of Endocrinology and Metabolism, Jiangmen Central Hospital, Jiangmen, Guangdong Province, China; 3 Department of Orthopedics, The Third Hospital of Xiamen, Xiamen, Fujian Province, China; 4 Division of Spine Surgery, Department of Orthopaedics, Nanfang Hospital, Southern Medical University, Guangzhou, Guangdong Province, China

**Keywords:** CCNL1, enhancer RNAs (eRNAs), immune signatures, multi-omics, osteoarthritis, type 2 diabetes mellitus

## Abstract

**Background:**

Osteoarthritis (OA) and type 2 diabetes mellitus (T2DM) frequently coexist and share inflammatory and metabolic disturbances, but the immune-epigenetic features that may overlap between these conditions remain incompletely defined. Enhancer RNAs (eRNAs) are increasingly recognized as regulators of enhancer activity and transcriptional programs, whereas eRNA-associated signatures shared between OA-related and T2DM-related datasets remain largely unexplored.

**Methods:**

Transcriptomic datasets from OA joint tissues and controls (63 OA, 35 controls) were analyzed together with an independent T2DM dataset of CD14^+^ monocytes (5 T2DM, 5 controls) to explore overlapping molecular features across distinct disease-related contexts. Differential expression analysis, exploratory Weighted Gene Co-expression Network Analysis (WGCNA), CIBERSORT-based estimation, and pathway analyses were used to prioritize eRNA-associated features, transcription factors (TFs), candidate genes, and immune-associated transcriptional patterns. Public ATAC-seq, ChIP-seq, and scRNA-seq datasets, together with a high-glucose stress model in rat chondrocytes, were used to provide epigenomic, cellular, and preliminary experimental context for the candidate associations.

**Results:**

We identified 473 annotated dysregulated eRNA-associated features in OA, with associated genes enriched in skeletal development, extracellular matrix remodeling, and PI3K-Akt signaling. CIBERSORT-based and pathway analyses suggested immune-associated transcriptional alterations and inflammatory pathway activation in OA. Integrative analysis prioritized TOB1 as a candidate eRNA-associated feature related to JUN, *CCNL1*, and inflammatory signaling within an inferred correlation-based network. Public ATAC-seq, ChIP-seq, and scRNA-seq datasets provided supportive epigenomic and cellular context for these candidate associations. Comparison with the T2DM monocyte dataset showed overlapping TNF-α/NF-κB and MAPK-related pathway alterations, with CCNL1 identified as a commonly downregulated candidate gene. *In vitro*, high-glucose stress was accompanied by reduced TOB1, JUN, and CCNL1 protein levels and increased TNF-α expression in chondrocytes.

**Conclusion:**

TOB1 was prioritized as a candidate eRNA-associated signal related to JUN, *CCNL1*, and inflammatory signaling across OA-related and T2DM-related datasets. These findings provide a bioinformatics-driven and correlation-based framework supported by public epigenomic context and preliminary *in vitro* data, offering potential directions for future studies of immune-metabolic overlap between OA and T2DM.

## 1 Introduction

Osteoarthritis (OA) is a prevalent degenerative joint disorder characterized by progressive articular cartilage breakdown and extracellular matrix (ECM) degradation, leading to pain and loss of joint function (Xie and Chen, 2019). Global estimates indicate that in 2020, OA affected approximately 595 million individuals, accounting for 14.8% of the population aged 30 years or older (Global and regional, 2023). OA pathogenesis has traditionally been attributed to excessive mechanical stress and impaired cartilage integrity (Messier et al., 2013). However, increasing evidence indicates that metabolic and inflammatory factors also contribute substantially to disease onset and progression (Wei et al., 2023; Zhang et al., 2025). Obesity and metabolic syndrome can promote chronic low-grade systemic inflammation, creating a detrimental microenvironment for joint tissues (Mocanu et al., 2024). Adipose tissue-derived adipokines and cytokines may promote cartilage breakdown and aberrant bone remodeling (Nedun et al., 2022). In addition, metabolic dysregulation may shift synovial macrophages toward a pro-inflammatory phenotype, stimulate matrix metalloproteinase (MMP) production, and amplify tissue injury (Jia et al., 2023; Veronese et al., 2019). Adaptive immune cells, including T helper cells, may further contribute to this inflammatory cascade (Li et al., 2017). These findings suggest that metabolic and inflammatory pathways may converge to shape OA progression.

Type 2 diabetes mellitus (T2DM) is another common chronic metabolic disorder that is epidemiologically and pathophysiologically associated with OA. Individuals with T2DM have a higher risk of developing OA, which may not be fully explained by body weight alone (Veronese et al., 2019). OA and T2DM share several biological features, including persistent low-grade inflammation, oxidative stress, and altered ECM metabolism (Piva et al., 2015). Despite these overlaps, the molecular features shared between OA-related joint pathology and T2DM-related systemic metabolic stress remain incompletely understood. Immune dysregulation may provide a relevant biological context for this overlap. Macrophages and T cells contribute to joint inflammation in OA, whereas hyperglycemia in T2DM can disrupt immune cell function and sustain systemic inflammatory activation (Berbudi et al., 2020). These shared immune-metabolic features highlight the need to identify overlapping molecular signatures associated with OA-related and T2DM-related disease contexts.

Epigenetic regulation, particularly enhancer-mediated transcriptional control, has emerged as an important mechanism underlying immune and metabolic responses. Enhancers are distal regulatory DNA elements that promote target gene transcription through interactions with gene promoters. In addition to serving as transcription factor (TF) binding platforms, active enhancers can be transcribed into non-coding RNAs known as enhancer RNAs (eRNAs) (Arnold et al., 2019). Emerging evidence suggests that eRNAs participate in transcriptional regulation by modulating chromatin accessibility, facilitating enhancer-promoter communication, and coordinating context-specific gene expression programs (Arnold et al., 2019). Because inflammatory and metabolic responses are tightly regulated at the transcriptional level, eRNA-associated features may provide a useful entry point for investigating immune-epigenetic dysregulation in OA. However, eRNA-associated molecular signatures shared between OA-related and T2DM-related datasets remain largely unexplored.

In this study, we aimed to characterize shared immune-epigenetic features across OA-related and T2DM-related datasets, with a focus on eRNA-associated transcriptional changes and correlation-based molecular networks. By integrating transcriptomic and multi-omics datasets, we prioritized differentially expressed eRNA-associated features, TFs, candidate genes, immune-associated transcriptional patterns, and disease-related signaling pathways. Public ChIP-seq, ATAC-seq, and scRNA-seq datasets were used to provide epigenomic and cellular context for the inferred candidate associations. Finally, a high-glucose stress model in rat chondrocytes was used to provide preliminary experimental context for whether the prioritized molecules showed stress-related expression changes. This integrative strategy prioritized TOB1, JUN, and CCNL1 as candidate molecules associated with immune-metabolic overlap between OA-related and T2DM-related datasets.

## 2 Materials and methods

### 2.1 Data acquisition and preprocessing

**FIGURE 1:**
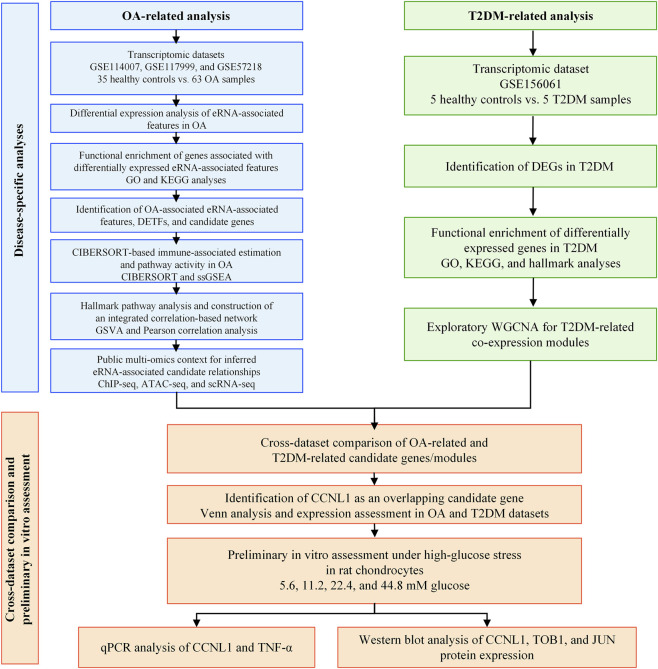
Schematic flowchart of the overall study design. The workflow summarizes the collection of transcriptomic datasets for osteoarthritis (OA) and type 2 diabetes mellitus (T2DM), followed by bioinformatic analyses including differential expression analysis, functional annotation, CIBERSORT-based immune-associated estimation, Gene Set Variation Analysis (GSVA), and exploratory Weighted Gene Co-expression Network Analysis (WGCNA). The study further included prioritization of overlapping candidate genes, construction of an inferred correlation-based network, and preliminary assessment using public multi-omics datasets and high-glucose stress-treated chondrocytes.

Transcriptomic datasets for osteoarthritis (OA), including GSE114007, GSE117999, and GSE57218, were downloaded from the Gene Expression Omnibus (GEO) database. To reduce batch effects, these datasets were merged and adjusted using the ComBat algorithm from the sva R package, resulting in a combined cohort of 35 healthy controls and 63 OA patients. The type 2 diabetes mellitus (T2DM) dataset (GSE156061), which included CD14^+^ monocytes from 5 diabetic patients and 5 healthy controls, was also obtained from GEO. The OA and T2DM datasets were analyzed as independent disease-related contexts to explore overlapping molecular features, rather than as a direct OA-T2DM comorbidity cohort. Transcription factor (TF) reference data (n = 318) were obtained from the Cistrome database (Taing et al., 2024). Immune-related genes and pathways were compiled from ImmPort (Bhattacharya et al., 2018) and the Molecular Signatures Database (MSigDB v7.0) (Subramanian et al., 2005; Liberzon et al., 2011; Liberzon et al., 2015). The overall study workflow is shown in Figure 1.

### 2.2 Identification of differentially expressed eRNA-associated features, TFs, and candidate genes

Annotated eRNA-associated features were obtained from eRNAbase, a curated database of enhancer RNA annotations (Song et al., 2024). The eRNAbase-derived eRNA list was matched to the expression matrix, and only eRNA-associated features represented on the selected transcriptomic platforms were retained for downstream analysis. Differential expression analysis between patients and healthy controls was performed using the ‘limma’ R package. Differentially expressed eRNA-associated features, transcription factors (TFs), and differentially expressed genes (DEGs) used as candidate genes in subsequent network analysis were identified using thresholds of false discovery rate (FDR) < 0.05 and |log2 fold change (FC)| > 1. Putative associations among differentially expressed TFs, differentially expressed eRNA-associated features, and DEGs used as candidate genes were inferred based on Pearson correlation analysis of their expression profiles. For the preliminary network between TFs and eRNA-associated features, associations with |r| > 0.300 and p < 0.05 were retained. For the more restricted candidate association screening integrating TFs, eRNA-associated features, candidate genes, and immune features, a threshold of |r| > 0.400 and p < 0.05 was applied. Network visualization was performed using Cytoscape. These relationships were treated as annotation-based and correlation-supported inferred associations. Functional enrichment analysis was performed on candidate genes associated with differentially expressed eRNA-associated features in the inferred correlation-based network using Gene Ontology (GO) and Kyoto Encyclopedia of Genes and Genomes (KEGG) pathway analyses.

### 2.3 Immune-associated transcriptional patterns and pathway activation analyses

The relative abundance of 22 immune cell subsets was computationally estimated from bulk transcriptomic profiles using the CIBERSORT algorithm. Single-sample gene set enrichment analysis (ssGSEA) was used to estimate the scores of 29 immune-related signatures. Gene Set Variation Analysis (GSVA) was used to evaluate hallmark signaling pathways. Non-parametric tests and Pearson correlation analyses (p < 0.05) were used to assess associations among clinical phenotypes, candidate eRNA-associated features, and immune-related signature scores. These analyses were used to infer immune-associated transcriptional patterns from bulk transcriptomic data and were not considered direct measurements of immune cell infiltration.

### 2.4 Integrated co-expression and correlation-based network construction

Pairwise correlations among differentially expressed TFs, differentially expressed eRNA-associated features, candidate genes, immune-related signatures, and hallmark pathways were calculated using Pearson correlation analysis. Associations meeting the thresholds of |r| > 0.300 and p < 0.001 were used to construct an integrated correlation-based network, which was visualized using Cytoscape (v3.7.1) (Shannon et al., 2003). Network edges were interpreted as statistical co-expression associations. The resulting correlation-based network was used to prioritize candidate molecular relationships for downstream contextual analyses. Complementary protein-protein interactions (PPIs) were predicted using the STRING database, retaining interactions with a confidence score >0.40 (Szklarczyk et al., 2023).

### 2.5 Epigenomic context assessment using ATAC-seq and ChIP-seq

Public JUN ChIP-seq and chromatin accessibility datasets were used to provide epigenomic context for candidate eRNA-associated regions. JUN ChIP-seq tracks were examined around the CCNL1 locus and the TOB1-related regulatory region. Public ATAC-seq tracks and ENCODE candidate cis-regulatory element annotations were used to evaluate chromatin accessibility and regulatory features around the TOB1-related region. These public epigenomic datasets provided contextual support for the inferred associations among TOB1, JUN, and CCNL1 (Taing et al., 2024; Moore et al., 2020; Lee et al., 2022; Buenrostro et al., 2013).

### 2.6 Exploratory WGCNA and candidate gene screening in T2DM

For the T2DM dataset (GSE156061), WGCNA was used to explore co-expression modules associated with T2DM status. Key parameters included a soft-thresholding power (β) selected to approximate scale-free topology and a minimum module size of 30 genes. Module eigengenes were correlated with T2DM status to identify candidate modules showing exploratory associations with disease status. Overlapping candidate genes between exploratory T2DM-related modules and OA-related candidate genes were identified using Venn intersection and further evaluated using non-parametric tests.

### 2.7 Single-cell RNA-seq analysis

Single-cell RNA sequencing (scRNA-seq) data from OA synovium (GSE152815) were analyzed to assess the cell-type distribution and co-expression patterns of candidate molecules at single-cell resolution. Cellular localization was further examined using the China National GeneBank Developmental Cell Platform (Li et al., 2022).

### 2.8 Rat chondrocyte isolation and high-glucose stress treatment

Primary chondrocytes were isolated from the knee joints of 10–14-day-old Sprague Dawley rats obtained from the Animal Experiment Center of Southern Medical University. Cartilage tissues were digested sequentially with 0.25% trypsin for 15 min and 0.2% collagenase II for 4 h at 37 °C. The extracted cells were cultured in DMEM/F12 supplemented with 10% FBS and 1% penicillin-streptomycin at 37 °C with 5% CO_2_. To establish high-glucose stress conditions *in vitro*, first-passage chondrocytes at 70% confluence were exposed to glucose gradients of 5.6 mM (normoglycemic control), 11.2 mM, 22.4 mM, and 44.8 mM using D-(+)-glucose for 48 h.

### 2.9 Cell viability and mannitol osmotic control assays

Cell viability and osmotic effects under different glucose concentrations were evaluated using a Cell Counting Kit-8 (CCK-8) assay and a mannitol osmotic control. Primary chondrocytes were seeded into 96-well plates at a density of 5 × 10^3^ cells/well and exposed to 5.6, 11.2, 22.4, or 44.8 mM glucose for 12, 24, 48, or 72 h. After treatment, CCK-8 reagent was added to each well and incubated for 1 h at 37 °C. Absorbance was recorded at 450 nm using an I3x multifunctional microplate reader. To control for osmotic effects, a parallel osmotic control group was treated with 5.6 mM glucose plus 39.2 mM mannitol for 48 h, matching the osmolarity of the 44.8 mM glucose stress condition. Cell viability was normalized to the 5.6 mM glucose control group.

### 2.10 Quantitative real-time PCR (qPCR)

Total RNA was extracted from chondrocytes using the SteadyPure RNA Extraction Kit (Accurate Biotechnology, China) and reverse-transcribed into cDNA using the Evo M-MLV RT Mix Kit. qPCR was performed on an Applied Biosystems StepOne Plus system using the SYBR Green Premix Pro Taq HS qPCR Kit. Relative mRNA expression was calculated using the 2^−ΔΔCt^ method and normalized to β-actin. The primer sequences were as follows: CCNL1 (F: 5′-GGG​CAG​GTG​TTG​TTT​CAT​CG-3′, R: 5′-GGG​GGC​TTG​GAG​TCC​TTT​TT-3′); TNF-α (F: 5′-CAC​CAC​GCT​CTT​CTG​TCT​ACT​G-3′, R: 5′-TGG​GCT​ACG​GGC​TTG​TCA​CT-3′).

### 2.11 Western blotting

Cells were lysed in RIPA buffer on ice, and total protein concentrations were quantified using a BCA assay kit (Beyotime, China). Equal amounts of protein (20 μg) were separated by SDS-PAGE and transferred to PVDF membranes. After blocking for 20 min (Protein Free Rapid Blocking Buffer, Epizyme, China), the membranes were incubated overnight at 4 °C with primary antibodies against CCNL1 (1:1,000, Huabio, ER63156), JUN (1:1,000, Proteintech, 24909-1-AP), TOB1 (1:1,000, Proteintech, 14915-1-AP), and β-actin (1:5000, Proteintech, 20536-1-AP). The membranes were then washed and incubated with an HRP-conjugated secondary antibody (1:50,000, Huabio, HA1001) for 1 h at room temperature. Protein bands were visualized using enhanced chemiluminescence (ECL, Beyotime).

### 2.12 Statistical analysis

Bioinformatics analyses were performed using R software (v4.3.1), and experimental data were analyzed using GraphPad Prism 10. Statistical procedures were applied consistently across comparable analyses. For bioinformatics analyses involving multiple comparisons, multiple testing correction was performed where applicable using the Benjamini–Hochberg method. Comparisons among multiple experimental groups, including CCK-8, qPCR, and Western blot assays across glucose gradients, were performed using one-way ANOVA followed by Tukey’s *post hoc* test. Comparisons involving the high-glucose stress group and the corresponding mannitol osmotic control were analyzed using one-way ANOVA with appropriate *post hoc* testing or two-tailed Student’s t-test when only two groups were compared. Experimental data are presented as mean ± standard deviation (SD) from three independent experiments (n = 3). Statistical significance was defined as p < 0.05.

## 3 Results

### 3.1 Dysregulated eRNA-associated transcriptional features in OA

**FIGURE 2:**
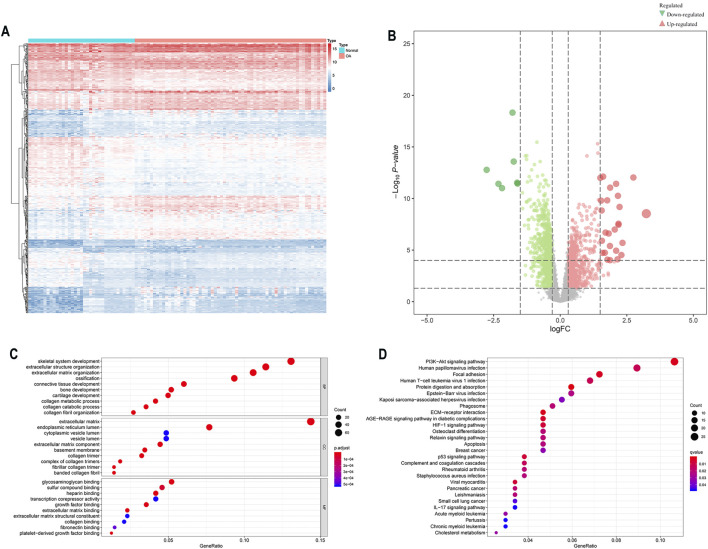
Identification of differentially expressed eRNA-associated features and functional annotation in OA. **(A)** Heatmap showing the expression profiles of differentially expressed eRNA-associated features between healthy controls and OA patients. **(B)** Volcano plot showing the distribution of upregulated and downregulated differentially expressed eRNA-associated features according to log2 fold change and statistical significance. **(C)** GO enrichment analysis of genes associated with differentially expressed eRNA-associated features, including Biological Process (BP), Cellular Component (CC), and Molecular Function (MF) categories. **(D)** KEGG pathway enrichment analysis of genes associated with the differentially expressed eRNA-associated features.

To characterize enhancer-associated transcriptional alterations in OA, we first compared annotated eRNA-associated expression features between healthy controls (n = 35) and patients with OA (n = 63). A total of 473 differentially expressed annotated eRNA-associated features were identified, and their expression patterns distinguished OA samples from healthy controls, suggesting broad enhancer-associated transcriptional alterations in OA (Figures 2A,B). GO analysis of genes linked to these annotated eRNA-associated features showed enrichment in biological processes and molecular functions related to skeletal system development, extracellular matrix organization, ossification, and glycosaminoglycan binding, suggesting that these features were associated with cartilage structural homeostasis-related processes in OA (Figure 2C). KEGG pathway analysis further revealed significant enrichment in the PI3K-Akt signaling pathway, along with pathways related to matrix remodeling, inflammation, and cell survival in OA (Figure 2D).

**FIGURE 3:**
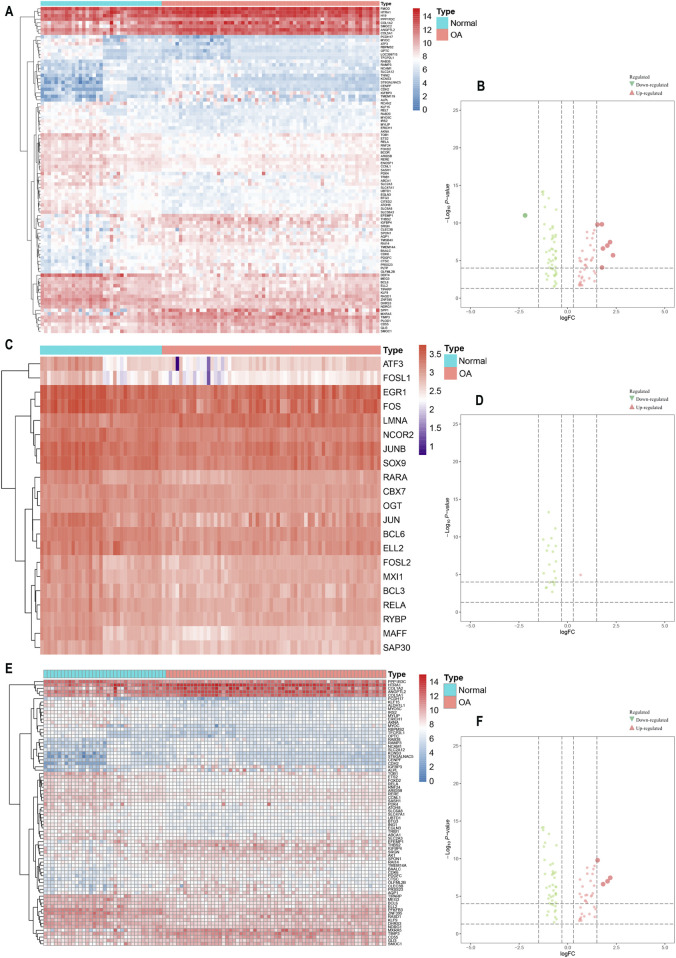
Expression profiles of OA-related eRNA-associated features, transcription factors (TFs), and candidate genes. **(A,B)** Heatmap **(A)** and volcano plot **(B)** showing the expression patterns of prioritized OA-related eRNA-associated features. **(C,D)** Heatmap **(C)** and volcano plot **(D)** displaying differentially expressed TFs between the groups. **(E,F)** Heatmap **(E)** and volcano plot **(F)** showing the differential expression landscape of the candidate genes. These findings summarize the expression patterns of candidate eRNA-associated transcriptional components in OA.

To further prioritize candidate OA-associated enhancer-related signals, we focused on 93 highly significant annotated eRNA-associated features from this differential set (Figures 3A,B). Based on these prioritized features, we identified 21 differentially expressed transcription factors (TFs) and 78 differentially expressed genes used as candidate genes for subsequent network analysis (Figures 3C–F). Most differentially expressed TFs were downregulated in OA, indicating a predominance of reduced TF expression among the eRNA-associated transcriptional components analyzed here. The candidate genes also showed distinct expression differences between healthy and OA samples. Together, these findings suggest that eRNA-associated transcriptional features are altered in OA.

### 3.2 Immune-associated transcriptional patterns and pathway activity in OA

**FIGURE 4:**
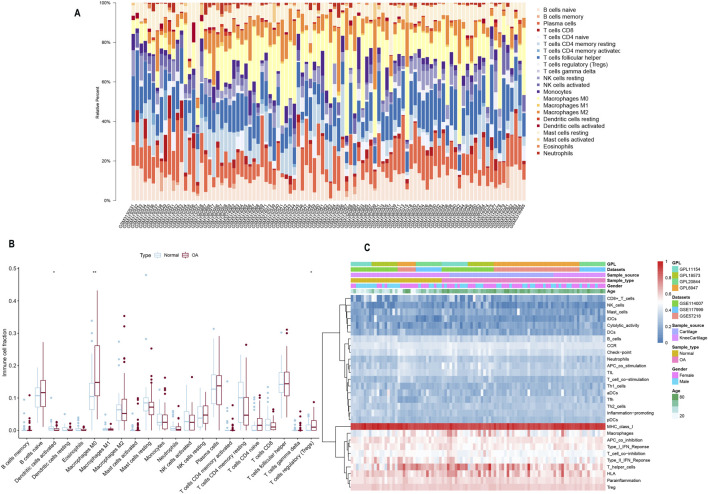
Characterization of immune-associated transcriptional patterns and pathway activity in OA. **(A)** Stacked bar chart illustrating the CIBERSORT-estimated relative proportions of 22 immune cell subsets in healthy and OA tissues. **(B)** Boxplots comparing differences in estimated immune cell proportions between the healthy control and OA groups. **(C)** Heatmap showing the single-sample gene set enrichment analysis (ssGSEA) scores, reflecting variations in immune-related signature scores and pathway features.

To explore immune-associated transcriptional patterns in OA, CIBERSORT was used to computationally estimate the relative abundance of 22 immune cell types from bulk transcriptomic profiles (Figure 4A). Among the estimated immune cell subsets, activated dendritic cells and M0 macrophages showed significant differences between OA and control samples (Figure 4B). These results indicate that OA tissues exhibit immune-associated transcriptional differences rather than changes attributable to a single estimated immune cell subset. In parallel, ssGSEA revealed heterogeneous alterations in immune-related signatures across OA and control samples (Figure 4C). Signatures related to macrophages, interferon responses, antigen presentation, T helper cells, parainflammation, and Treg-related activity showed distinct expression patterns, supporting the presence of immune-associated transcriptional alterations in OA. Together, these results suggest that estimated immune-associated transcriptional patterns and inflammatory pathway activity are altered in OA tissues.

### 3.3 Hallmark signaling pathways and an inferred correlation-based network in OA

**FIGURE 5:**
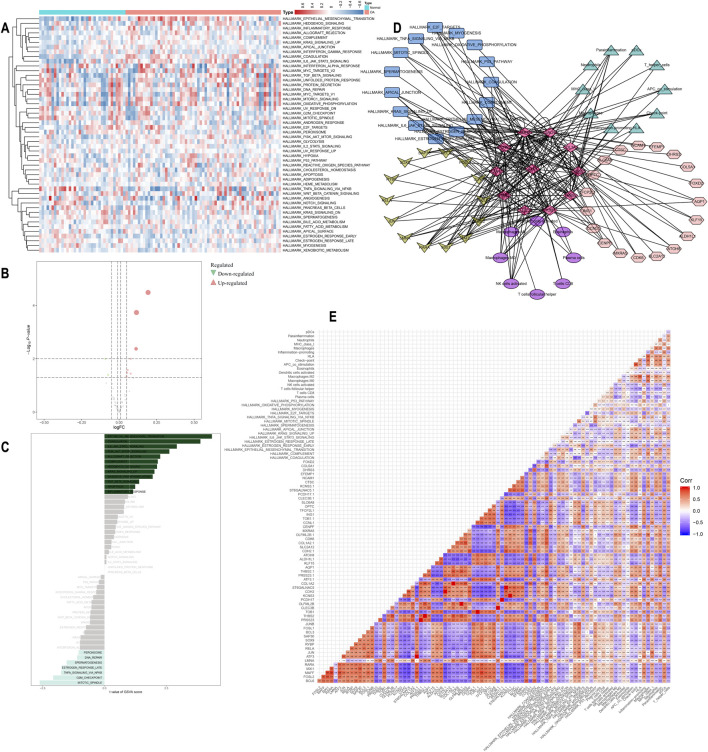
Identification of aberrant hallmark pathways and construction of an inferred correlation-based network. **(A,B)** Heatmap **(A)** and volcano plot **(B)** depicting the differential activity of hallmark gene sets between normal and OA samples, calculated using GSVA. **(C)** Bar plot ranking the differentially activated hallmark pathways based on their t-values. **(D)** Integrated correlation-based network showing associations among eRNA-associated features (diamonds), TFs (V-shapes), candidate genes/protein-related nodes (hexagons), estimated immune cell subsets (triangles), immune pathways (ovals), and hallmark pathways (rectangles). **(E)** Heatmap showing the Pearson correlation matrix among the network components, highlighting correlation-based associations in OA.

GSVA revealed distinct activity patterns of hallmark gene sets between OA and control samples (Figure 5A), with 19 of the 50 hallmark pathways showing significant differential activity (Figures 5B,C). To explore potential associations between pathway-level alterations and transcriptional components, we constructed an integrated correlation-based network using the filtered differentially expressed TFs and candidate genes based on Pearson correlation analysis (|r| > 0.300, p < 0.001) (Figure 5D). Within this network, JUN and CCNL1 showed the strongest correlation (r = 0.740, p < 0.001), indicating a close co-expression association between these two candidate components. Further mapping of 406 correlation-based associations prioritized 11 differentially expressed eRNA-associated features as enhancer-related candidate components. Among them, TOB1, a candidate eRNA-associated feature, showed strong co-expression with *CCNL1* (r = 0.740, p < 0.001), suggesting a candidate association between TOB1 and *CCNL1* in OA. Correlation analysis also showed that *CCNL1* was negatively associated with coagulation (r = −0.60, p < 0.001), epithelial-mesenchymal transition (r = −0.47, p < 0.001), and IL6-JAK-STAT3 signaling (r = −0.45, p < 0.001) (Figure 5E).

To further examine the epigenomic context of these inferred associations, we analyzed public ChIP-seq and chromatin accessibility datasets. Public JUN ChIP-seq tracks showed binding signals around the CCNL1 locus, providing epigenomic context for this candidate association (Supplementary Figure S1). Public JUN ChIP-seq signals were also observed around the TOB1-related regulatory region. In addition, imputed ATAC-seq signals from bone marrow mesenchymal stem cells, experimental ATAC-seq signals from IMR-90 fibroblast-related cells, and cCRE annotations indicated an accessible chromatin context at this locus (Supplementary Figure S1). Together, these public epigenomic datasets provide complementary context for the inferred association among TOB1, JUN, and CCNL1.

We next examined the cellular distribution of these candidate molecules using scRNA-seq data from OA synovium. Nine fibroblast clusters were identified (Supplementary Figure S2), with marker profiles related to immune regulation, fibrotic remodeling, and pro-inflammatory signaling (Supplementary Figure S2). Feature plots and co-localization analyses showed overlapping expression patterns among TOB1, JUN, and CCNL1 in fibroblast populations (Supplementary Figure S2). Specifically, 35% of cells co-expressed TOB1 and CCNL1, whereas 50% co-expressed JUN and CCNL1, suggesting that these candidate molecules show overlapping transcriptomic distribution within fibroblast-rich cellular niches. Interestingly, Cluster 8 showed limited expression of these candidate molecules (Supplementary Figure S2 but high expression of LGALS1, a marker associated with fibroblast phenotypic remodeling (Supplementary Figure S2) (Osório, 2016).

### 3.4 Gene expression and exploratory co-expression analysis in T2DM

**FIGURE 6:**
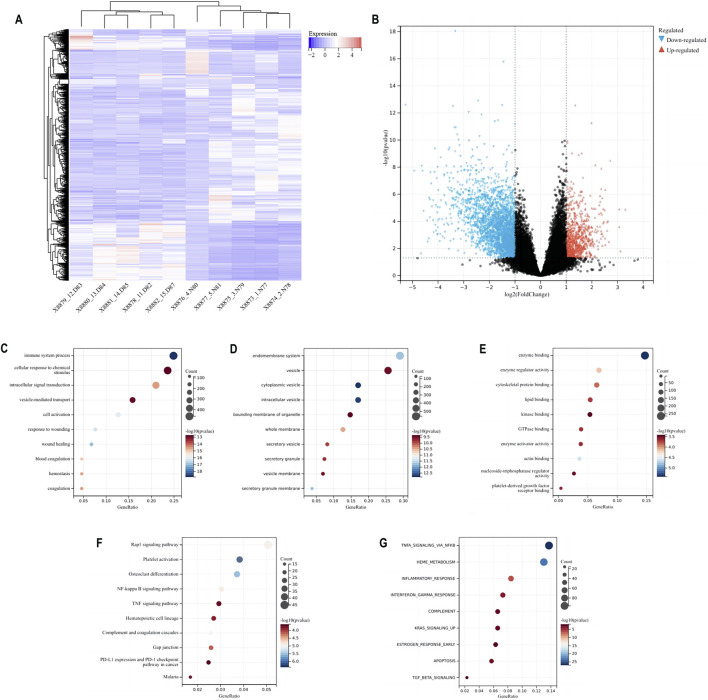
Differential expression and functional enrichment analyses in the T2DM dataset. **(A)** Heatmap visualizing the differentially expressed genes (DEGs) in samples from T2DM patients and healthy controls. **(B)** Volcano plot showing the distribution of upregulated and downregulated DEGs. **(C–E)** Bubble plots of GO enrichment analysis showing the top enriched terms in Biological Process **(C)**, Cellular Component **(D)**, and Molecular Function **(E)**. **(F)** KEGG pathway enrichment analysis of the identified DEGs. **(G)** Hallmark gene set enrichment analysis highlighting TNF-α signaling via NF-κB and related pathways among the prominent signatures in T2DM.

To determine whether the T2DM dataset shared molecular features with OA, we analyzed the GSE156061 dataset, which profiles CD14^+^ monocytes from patients with T2DM and healthy controls. Monocytes are important contributors to systemic inflammation and therefore provide a relevant cellular context for exploring T2DM-related inflammatory transcriptional features. Differential expression analysis identified 5970 dysregulated genes between T2DM and control samples (Figures 6A,B). Functional enrichment analysis highlighted immune responses and endomembrane system-related processes (Figures 6C–E), whereas KEGG and hallmark analyses emphasized Rap1 signaling and TNF-α/NF-κB-related inflammatory pathways (Figures 6F,G).

**FIGURE 7:**
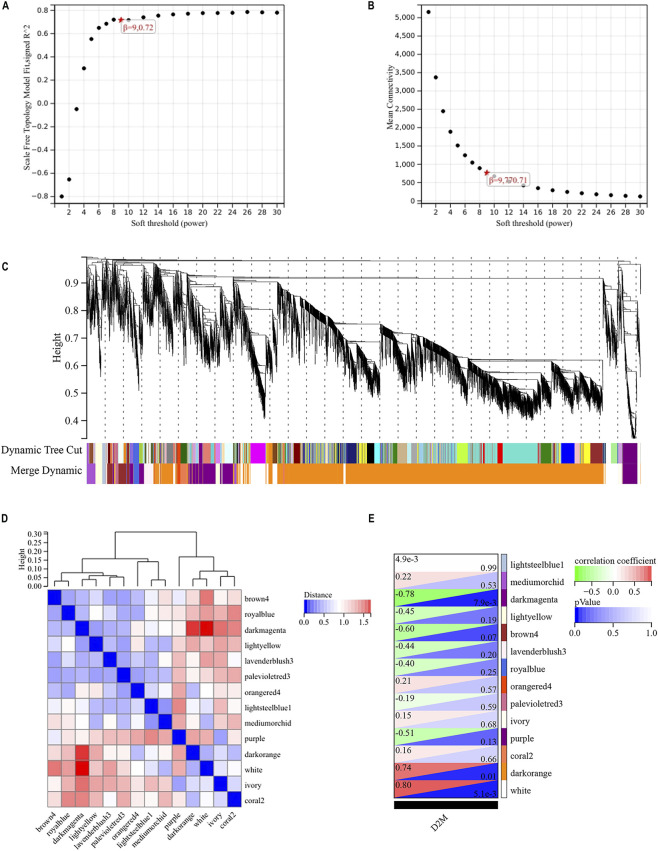
Exploratory Weighted Gene Co-expression Network Analysis (WGCNA) of the T2DM dataset. **(A,B)** Analysis of the scale-free fit index **(A)** and mean connectivity **(B)** across different soft-thresholding powers to establish an approximate scale-free network topology. **(C)** Hierarchical clustering dendrogram of genes, with distinct co-expression modules assigned different colors (Dynamic Tree Cut). **(D)** Heatmap showing the hierarchical clustering and topological overlap (distance) among the identified modules. **(E)** Heatmap of module–trait relationships evaluating the correlations between specific gene modules and T2DM status, identifying the darkmagenta module as showing the strongest exploratory association.

Exploratory WGCNA identified 14 co-expression modules in the T2DM dataset (Figures 7A–C). Within this exploratory analysis, the darkmagenta module showed the strongest negative correlation with T2DM status (r = −0.78, p < 0.01) (Figures 7C–E). Pathway analysis of the 611 candidate genes within this module indicated enrichment of MAPK signaling-related pathways (Supplementary Figure S3). Notably, several dysregulated pathways in T2DM, including TNF-α/NF-κB and MAPK signaling, overlapped with OA-associated pathway alterations, suggesting partially overlapping pathway-level signals between OA tissue datasets and the T2DM monocyte dataset.

### 3.5 Overlapping candidate gene screening and preliminary *in vitro* assessment

**FIGURE 8:**
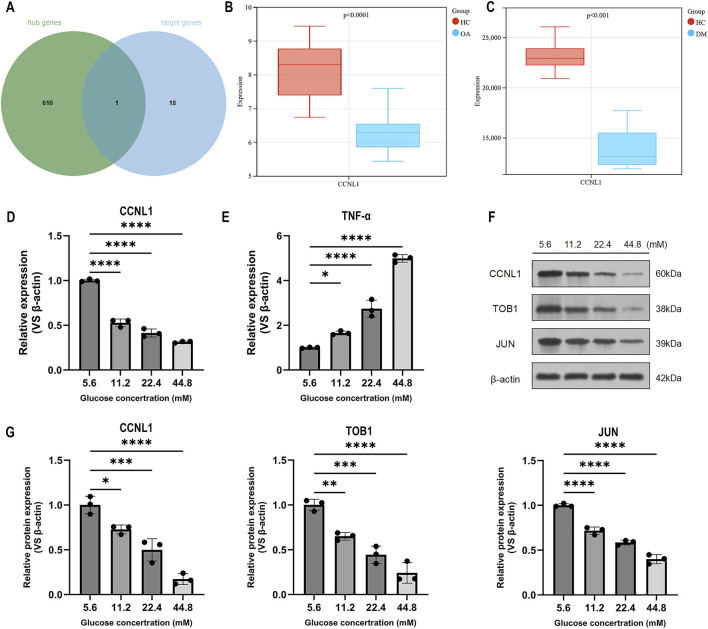
Preliminary *in vitro* assessment of CCNL1 and candidate molecules related to TOB1, JUN, and CCNL1 under high-glucose stress conditions. **(A)** Venn diagram identifying CCNL1 as an overlapping candidate gene between OA-related and T2DM-related candidate gene sets. **(B,C)** Boxplots showing CCNL1 expression in the OA dataset (B, GSE114007) and the T2DM monocyte dataset (C, GSE156061). **(D,E)** Quantitative real-time PCR (qPCR) analysis showing the relative mRNA expression levels of CCNL1 **(D)** and the inflammatory marker TNF-α **(E)** in chondrocytes exposed to increasing glucose stress conditions (5.6, 11.2, 22.4, and 44.8 mM). **(F)** Representative Western blot images showing the protein levels of CCNL1, TOB1, and JUN after exposure to increasing glucose concentrations. **(G)** Densitometric quantification of CCNL1, TOB1, and JUN protein levels, normalized to β-actin. Data are presented as mean ± SD from three independent experiments (n = 3). Statistical significance was evaluated using one-way ANOVA followed by Tukey’s *post hoc* test; *p < 0.05, **p < 0.01, ***p < 0.001, ****p < 0.0001.

Venn diagram analysis of OA-related and T2DM-related candidate gene sets identified CCNL1 as an overlapping candidate gene between the two disease-related contexts (Figure 8A). Consistently, CCNL1 was significantly downregulated in both the OA cohort (p < 0.001) (Figure 8B) and the T2DM monocyte cohort (p < 0.01) (Figure 8C), suggesting that CCNL1 may represent a cross-dataset overlapping candidate signal.

To define a high-glucose stress condition for subsequent preliminary *in vitro* assessment, primary SD rat chondrocytes were exposed to increasing glucose concentrations for different durations. CCK-8 analysis showed that cell viability declined in a concentration- and time-dependent manner (Supplementary Figure S4), and 44.8 mM glucose for 48 h was used as the high-glucose stress condition for subsequent preliminary stress-response assessment. To distinguish glucose-associated effects from osmotic effects, a mannitol osmotic control was included. The mannitol control indicated that the observed viability changes were not attributable to osmotic effects alone (Supplementary Figure S4). Following this viability and osmotic control assessment, qPCR analysis showed that CCNL1 mRNA expression decreased under the high-glucose stress condition (p < 0.001) (Figure 8D). In contrast, TNF-α expression increased progressively in response to rising glucose concentrations, reaching the highest level at 44.8 mM (p < 0.001) (Figure 8E).

Western blot analysis further assessed the protein-level changes of candidate molecules identified from the inferred network. The protein levels of CCNL1, TOB1, and JUN progressively declined with increasing glucose concentrations (Figure 8F). Quantitative analysis showed significant downregulation of all three proteins under the high-glucose stress condition (p < 0.001) (Figure 8G). Together, these findings suggest that the changes in TOB1, JUN, and CCNL1 protein levels and inflammatory marker expression are consistent with a high-glucose stress response in chondrocytes.

## 4 Discussion

OA is increasingly recognized as a disease influenced by both local joint pathology and systemic metabolic disturbances (Courties et al., 2017). Accumulating evidence suggests that OA and T2DM share inflammatory and metabolic features, although the molecular overlap between these conditions remains incompletely defined (Seow et al., 2024). Both diseases have been associated with chronic low-grade inflammation, oxidative stress, and altered extracellular matrix metabolism, providing a rationale for exploring shared molecular signatures across OA-related and T2DM-related datasets (Mendes et al., 2015). However, the overlapping molecular signatures between OA-related joint tissues and T2DM-related immune cells remain insufficiently characterized (Courties et al., 2019). In this study, we prioritized TOB1, JUN, and *CCNL1* as candidate molecules within an inferred correlation-based network related to OA-associated inflammatory and transcriptional features. Rather than supporting a linear regulatory cascade, our findings suggest that these molecules may represent coordinated components of a broader transcriptional response. Their parallel protein-level changes under high-glucose stress are consistent with a high-glucose-related stress response in chondrocytes, rather than establishing a specific regulatory mechanism.

The immune-associated transcriptional patterns observed in this study provide contextual information for interpreting OA-related inflammatory signatures. Our analysis suggested immune-associated transcriptional differences in OA, including estimated changes in activated dendritic cells and M0 macrophages, together with alterations in TNF-related, interferon, and IL6-JAK-STAT3 pathway signatures (Wiegertjes et al., 2020; Latourte et al., 2017). These findings are consistent with previous studies showing that OA is accompanied by chronic immune activation and cytokine-driven inflammation that contributes to synovial inflammation and cartilage degeneration (Sanchez-Lopez et al., 2022). However, most prior studies have described these immune alterations at the level of cell composition or signaling pathways, with limited insight into their association with broader transcriptional changes.

The coordinated activation of multiple inflammatory pathways observed here is consistent with the possibility that transcriptional programs may be associated with these responses. In line with this observation, our network analysis found a strong co-expression association between JUN and *CCNL1*. *CCNL1* appears to represent a candidate molecule within the inferred correlation-based network, given its role in maintaining transcriptional homeostasis and cell cycle regulation (Loyer and Trembley, 2020). Its consistent downregulation may reflect broader transcriptional changes associated with OA-related inflammatory and metabolic stress. As a stress-responsive transcription factor, JUN integrates inflammatory cues and may participate in coordinating transcriptional responses (Schonthaler et al., 2011). Therefore, altered JUN-related activity may coincide with transcriptional imbalance and reduced *CCNL1* expression. In rheumatoid arthritis, JUN is involved in pathogenic fibroblast activation and promotes inflammatory signaling through interactions with YAP, PTPN14, and SMAD3 pathways (Bottini et al., 2019). In contrast, JUN expression has been reported to be reduced in aging-related synovial tissues in OA, and lower JUN-related activity has been linked to enhanced immune-associated signatures and chondrocyte senescence (Zhou et al., 2023; Xie et al., 2023). Collectively, these findings suggest that altered JUN-related transcriptional activity may be associated with OA-related immune and transcriptional dysregulation. Such changes may coincide with altered expression of candidate genes, including *CCNL1*, and enhanced inflammatory signaling.

Network analysis further highlighted TOB1 as an enhancer-related candidate associated with *CCNL1*. The co-expression between TOB1 and *CCNL1*, together with public JUN ChIP-seq signals around the TOB1-related regulatory region, provides contextual support for considering TOB1 as an enhancer-related candidate associated with *CCNL1*. In addition, imputed ATAC-seq signals from bone marrow mesenchymal stem cells, experimental ATAC-seq signals from IMR-90 fibroblast-related cells, and cCRE annotations indicated that this region is located within an accessible regulatory landscape. These findings suggest that TOB1 may represent an inferred enhancer-associated signal in OA, although its direct eRNA activity remains to be experimentally validated. TOB1, a member of the Tob/BTG anti-proliferative protein family, has also been implicated in cartilage homeostasis at the gene and protein levels. These gene- and protein-level observations provide biological context for the TOB1 locus, while the functional relevance of TOB1 eRNA itself remains to be clarified. Gebauer et al. demonstrated that TOB1 is significantly downregulated in OA chondrocytes, reaching approximately one-sixth of normal levels. This reduction is associated with increased proliferation markers such as Ki-67 and altered expression of differentiation markers including COL2A1, suggesting disruption of the proliferation-differentiation balance required for chondrocyte stability (Gebauer et al., 2005). Reduced TOB1 expression may therefore be associated with impaired cartilage homeostasis, abnormal proliferation, metabolic imbalance, and matrix degradation. Beyond joint pathology, TOB1 has also been shown to exert anti-inflammatory effects by mediating Th1/Th17 differentiation through the Smad4/5-ID2 axis (Lin et al., 2022). Within this context, the TOB1 locus may represent a biologically relevant region where enhancer-associated signals, cartilage-related transcriptional changes, and immune-related features converge. The relationship between TOB1 protein expression and TOB1 eRNA activity remains to be clarified, as does the potential regulatory relevance of TOB1 eRNA to CCNL1 expression or inflammatory signaling.

CCNL1 appears to represent an important candidate molecule within the inferred correlation-based network. CCNL1 is involved in transcriptional homeostasis, RNA splicing, and cell cycle regulation. Through interactions with CDK11-related machinery, CCNL1 is functionally linked to transcriptional and splicing regulation (Loyer and Trembley, 2020). Its consistent downregulation in the analyzed datasets may therefore reflect broader transcriptional alterations associated with OA-related and T2DM-related inflammatory contexts. Previous bioinformatic analyses have identified CCNL1 among OA-associated inflammatory gene signatures (Li et al., 2023). These findings suggest that CCNL1 may be associated with OA-related inflammatory regulation rather than merely serving as a passive marker. CCNL1 may also be connected to metabolic disease-related regulation. Genetic studies have identified variants near the CCNL1/LEKR1 locus that are associated with diabetic retinopathy susceptibility (L et al., 2016). These observations are consistent with the possibility that CCNL1 may be related to inflammatory and metabolic transcriptional contexts. Taken together, CCNL1 may represent a candidate molecule associated with TOB1 and JUN within the inferred correlation-based network.

Building on this framework, our findings suggest that metabolic stress may be associated with changes in this inferred molecular pattern. Cross-dataset analysis identified *CCNL1* as an overlapping dysregulated candidate gene in OA tissue and T2DM monocyte datasets, indicating that overlapping molecular features may be present across these disease-related contexts. Consistent with this possibility, our *in vitro* high-glucose stress experiments showed that increasing glucose concentrations were associated with parallel reductions in JUN, TOB1, and CCNL1 protein levels, accompanied by elevated TNF-α expression. These findings align with previous studies showing that high-glucose exposure promotes catabolic and inflammatory responses in chondrocytes (Liang et al., 2019) and that T2DM is characterized by chronic inflammatory activation (Nedosugova et al., 2022). These results suggest that high-glucose-related stress is accompanied by parallel changes in TOB1, JUN, and CCNL1 protein levels and enhanced inflammatory signaling in chondrocytes. Within this context, these molecules may serve as candidates for future studies examining how high-glucose-related stress influences OA-associated inflammatory responses.

Several limitations should be acknowledged. This study did not include a patient cohort with both OA and T2DM, and the integration of OA joint tissue datasets with a T2DM monocyte dataset may introduce biological and technical heterogeneity. Therefore, the overlapping molecular features identified here should be interpreted with caution. The T2DM dataset also included only five patients and five controls, which may limit the stability of the WGCNA-derived modules and overlapping candidate genes; these findings require validation in larger independent T2DM datasets. Another limitation is that the experimental assays measured TOB1 protein rather than TOB1 eRNA itself, and enhancer activity was not directly assessed using eRNA knockdown, enhancer reporter assays, or chromatin interaction assays. Similarly, CIBERSORT and ssGSEA were applied to bulk transcriptomic datasets and therefore provided computational estimates of immune-associated transcriptional patterns rather than direct measurements of immune cell infiltration; future studies using histology, flow cytometry, or spatial transcriptomics would help validate these immune-related findings. In addition, although the high-glucose stress model in rat chondrocytes provided preliminary experimental context, it differs from the human joint tissue and CD14^+^ monocyte datasets used in the bioinformatic analyses. OA and T2DM are complex conditions, and molecular patterns beyond the TOB1-related candidate signal are likely to be involved. Future studies should track CCNL1 expression during disease progression and determine whether targeted perturbation of CCNL1 or *TOB1* eRNA affects disease-related phenotypes. eRNA-specific validation and functional assays will also be necessary to clarify the regulatory relevance of *TOB1* eRNA and the biological or translational significance of these candidate molecules in OA-related and T2DM-related inflammatory contexts.

## 5 Conclusion

This study prioritized *TOB1* as a candidate eRNA-associated signal related to JUN, CCNL1, and inflammatory signaling in OA, and identified CCNL1 as an overlapping candidate gene in OA tissue and T2DM monocyte datasets. The correlation-based association among these molecules, together with their stress-related expression changes under high-glucose stress and concomitant inflammatory marker induction, suggests that they may be related to the molecular overlap between OA-related and T2DM-related contexts. These findings provide a bioinformatics-driven and correlation-based framework supported by public epigenomic context and preliminary *in vitro* data, offering potential directions for future studies of immune-metabolic overlap between OA and T2DM.

## Data Availability

The datasets analyzed in this study are publicly available from the Gene Expression Omnibus (GEO) and ENCODE databases. The OA transcriptomic datasets included GSE114007, GSE117999, and GSE57218. The T2DM monocyte transcriptomic dataset was GSE156061, and the scRNA-seq dataset used for single-cell analysis was GSE152815. Public JUN ChIP-seq data used to assess JUN binding around the CCNL1 locus and the TOB1-related regulatory region were obtained from GEO under accession number GSM487425 and accessed through the Cistrome Data Browser. Chromatin accessibility evidence was obtained from ENCODE, including imputed ATAC-seq signals from bone marrow mesenchymal stem cells under accession ENCSR075ELA and experimental ATAC-seq signals from IMR-90 cells under file accession ENCF770EAV. Candidate cis-regulatory element annotations were obtained from ENCODE cCRE tracks. No new sequencing data were generated in this study.
