# Supplementary material for: Integrated multi-omics analysis identifies candidate eRNA-associated signatures shared between osteoarthritis and type 2 diabetes
Source: Front Genet. 2026 Jul 17;17:1875546. doi: 10.3389/fgene.2026.1875546 (PMC13423288; doi:10.3389/fgene.2026.1875546)
Supplement: Supplementary file 1 [file Table1.docx]

**Table S1. Hub genes in the darkmagenta module identified by WGCNA analysis of the GSE156061 dataset.**

| Gene |  | Module |  | | MM_R |  | MM_pvalue |
| --- | --- | --- | --- | --- | --- | --- | --- |
| LOC729737 |  | darkmagenta |  | 0.883267867 | |  | 0.000703994 |
| LOC100133331 |  | darkmagenta |  | 0.874312897 | |  | 0.000935594 |
| RP4-669L17.10 |  | darkmagenta |  | 0.874305848 | |  | 0.000935796 |
| LOC100288069 |  | darkmagenta |  | 0.928953179 | |  | 0.000102245 |
| NADK |  | darkmagenta |  | 0.853639839 | |  | 0.001676025 |
| PHF13 |  | darkmagenta |  | 0.965933789 | |  | 5.65E-06 |
| UBE4B |  | darkmagenta |  | 0.855508146 | |  | 0.001595844 |
| KIF1B |  | darkmagenta |  | 0.867159534 | |  | 0.001157001 |
| RCC2 |  | darkmagenta |  | -0.89431211 | |  | 0.000479631 |
| ECE1 |  | darkmagenta |  | 0.955425655 | |  | 1.64E-05 |
| RP5-1071N3.1 |  | darkmagenta |  | 0.898998052 | |  | 0.000402405 |
| ALPL |  | darkmagenta |  | 0.919921852 | |  | 0.000163184 |
| EPHB2 |  | darkmagenta |  | -0.819897823 | |  | 0.00368103 |
| SEPN1 |  | darkmagenta |  | -0.868483404 | |  | 0.001113421 |
| ZDHHC18 |  | darkmagenta |  | 0.944484882 | |  | 3.89E-05 |
| SLC9A1 |  | darkmagenta |  | 0.90128069 | |  | 0.000368288 |
| THEMIS2 |  | darkmagenta |  | 0.892435698 | |  | 0.000513407 |
| XKR8 |  | darkmagenta |  | 0.891435342 | |  | 0.000532108 |
| SESN2 |  | darkmagenta |  | 0.912463232 | |  | 0.000230873 |
| RNF19B |  | darkmagenta |  | 0.820334664 | |  | 0.003647491 |
| STK40 |  | darkmagenta |  | 0.867476233 | |  | 0.001146465 |
| CSF3R |  | darkmagenta |  | 0.972437715 | |  | 2.44E-06 |
| ZC3H12A |  | darkmagenta |  | 0.879718144 | |  | 0.000790086 |
| MTF1 |  | darkmagenta |  | 0.803787959 | |  | 0.00507907 |
| RLF |  | darkmagenta |  | 0.851372767 | |  | 0.00177719 |
| PLK3 |  | darkmagenta |  | 0.925131181 | |  | 0.000125493 |
| BTBD19 |  | darkmagenta |  | 0.961575415 | |  | 9.10E-06 |
| MKNK1 |  | darkmagenta |  | 0.807223884 | |  | 0.004753585 |
| PDE4B |  | darkmagenta |  | 0.844412361 | |  | 0.002115439 |
| CTBS |  | darkmagenta |  | 0.934381863 | |  | 7.49E-05 |
| PKN2 |  | darkmagenta |  | 0.907154597 | |  | 0.000290264 |
| TAF13 |  | darkmagenta |  | 0.897495865 | |  | 0.000426085 |
| GNAI3 |  | darkmagenta |  | 0.871233197 | |  | 0.001026732 |
| AMPD2 |  | darkmagenta |  | 0.854563108 | |  | 0.001636045 |
| FAM212B |  | darkmagenta |  | 0.84901 | |  | 0.001887255 |
| PHTF1 |  | darkmagenta |  | 0.885212163 | |  | 0.000659855 |
| FCGR1B |  | darkmagenta |  | 0.846890869 | |  | 0.001990092 |
| FCGR1A |  | darkmagenta |  | 0.816254355 | |  | 0.003969433 |
| TUFT1 |  | darkmagenta |  | 0.886645439 | |  | 0.000628638 |
| S100A11 |  | darkmagenta |  | 0.914162605 | |  | 0.000213911 |
| SEMA4A |  | darkmagenta |  | 0.885884311 | |  | 0.000645078 |
| SLC25A44 |  | darkmagenta |  | 0.926710739 | |  | 0.000115458 |
| FCGR2A |  | darkmagenta |  | 0.928346259 | |  | 0.000105704 |
| RC3H1 |  | darkmagenta |  | 0.856953123 | |  | 0.001535766 |
| RFWD2 |  | darkmagenta |  | 0.977161423 | |  | 1.16E-06 |
| CACNA1E |  | darkmagenta |  | 0.966439479 | |  | 5.33E-06 |
| GLUL |  | darkmagenta |  | 0.941599535 | |  | 4.74E-05 |
| NCF2 |  | darkmagenta |  | 0.865414294 | |  | 0.001216331 |
| FAM129A |  | darkmagenta |  | 0.983663722 | |  | 3.06E-07 |
| RGS2 |  | darkmagenta |  | 0.806595227 | |  | 0.00481199 |
| KDM5B |  | darkmagenta |  | 0.848713221 | |  | 0.00190142 |
| LINC01136 |  | darkmagenta |  | 0.933151044 | |  | 8.06E-05 |
| PPP1R15B |  | darkmagenta |  | 0.838179266 | |  | 0.002455779 |
| CR1 |  | darkmagenta |  | 0.933977619 | |  | 7.67E-05 |
| CD46 |  | darkmagenta |  | 0.865603735 | |  | 0.001209786 |
| H3F3A |  | darkmagenta |  | 0.888115339 | |  | 0.000597758 |
| H3F3AP4 |  | darkmagenta |  | 0.888090365 | |  | 0.000598274 |
| EGLN1 |  | darkmagenta |  | 0.902897648 | |  | 0.000345439 |
| SIPA1L2 |  | darkmagenta |  | 0.929462227 | |  | 9.94E-05 |
| AHCTF1 |  | darkmagenta |  | 0.839332738 | |  | 0.002390016 |
| MSRB1 |  | darkmagenta |  | 0.833653828 | |  | 0.002726439 |
| MMP25 |  | darkmagenta |  | 0.984357108 | |  | 2.57E-07 |
| CREBBP |  | darkmagenta |  | 0.906268956 | |  | 0.000301167 |
| MGRN1 |  | darkmagenta |  | 0.945761814 | |  | 3.55E-05 |
| UBN1 |  | darkmagenta |  | 0.952006107 | |  | 2.19E-05 |
| C16orf72 |  | darkmagenta |  | 0.80564875 | |  | 0.004900887 |
| LITAF |  | darkmagenta |  | 0.979518359 | |  | 7.51E-07 |
| KIAA0556 |  | darkmagenta |  | 0.878098182 | |  | 0.000831824 |
| XPO6 |  | darkmagenta |  | 0.987117708 | |  | 1.19E-07 |
| APOBR |  | darkmagenta |  | 0.962605456 | |  | 8.18E-06 |
| PPP4C |  | darkmagenta |  | 0.805852568 | |  | 0.004881645 |
| MAPK3 |  | darkmagenta |  | 0.865345443 | |  | 0.001218716 |
| TBC1D10B |  | darkmagenta |  | 0.941821397 | |  | 4.67E-05 |
| PRR14 |  | darkmagenta |  | 0.8967544 | |  | 0.000438143 |
| FBRS |  | darkmagenta |  | 0.935362009 | |  | 7.06E-05 |
| ITGAX |  | darkmagenta |  | 0.95837655 | |  | 1.25E-05 |
| ZNF267 |  | darkmagenta |  | 0.887314652 | |  | 0.000614438 |
| N4BP1 |  | darkmagenta |  | 0.868562622 | |  | 0.001110851 |
| NOD2 |  | darkmagenta |  | 0.805485633 | |  | 0.004916325 |
| LOC643802 |  | darkmagenta |  | 0.896436151 | |  | 0.000443394 |
| GPR97 |  | darkmagenta |  | 0.968578486 | |  | 4.11E-06 |
| USB1 |  | darkmagenta |  | 0.843430024 | |  | 0.002166663 |
| CMTM2 |  | darkmagenta |  | 0.870016361 | |  | 0.001064463 |
| NFAT5 |  | darkmagenta |  | 0.854579749 | |  | 0.001635331 |
| WWP2 |  | darkmagenta |  | 0.903713162 | |  | 0.000334319 |
| ST3GAL2 |  | darkmagenta |  | 0.857725434 | |  | 0.001504338 |
| AP1G1 |  | darkmagenta |  | 0.936115591 | |  | 6.74E-05 |
| PLCG2 |  | darkmagenta |  | 0.929504879 | |  | 9.92E-05 |
| MAP1LC3B |  | darkmagenta |  | 0.854476675 | |  | 0.001639759 |
| ANKRD11 |  | darkmagenta |  | 0.894957247 | |  | 0.000468404 |
| VPS9D1 |  | darkmagenta |  | 0.847759735 | |  | 0.00194745 |
| DEF8 |  | darkmagenta |  | 0.849810869 | |  | 0.001849411 |
| LINC01002 |  | darkmagenta |  | 0.91618888 | |  | 0.000194905 |
| CNN2 |  | darkmagenta |  | 0.865326113 | |  | 0.001219386 |
| SBNO2 |  | darkmagenta |  | 0.830682658 | |  | 0.00291542 |
| MKNK2 |  | darkmagenta |  | 0.917039851 | |  | 0.000187306 |
| PLIN4 |  | darkmagenta |  | 0.913933865 | |  | 0.000216139 |
| PLIN5 |  | darkmagenta |  | 0.860435799 | |  | 0.001397724 |
| LRG1 |  | darkmagenta |  | 0.807843014 | |  | 0.004696561 |
| KDM4B |  | darkmagenta |  | 0.946404371 | |  | 3.38E-05 |
| MYO1F |  | darkmagenta |  | 0.954175557 | |  | 1.83E-05 |
| ICAM1 |  | darkmagenta |  | 0.826984761 | |  | 0.003163572 |
| ICAM3 |  | darkmagenta |  | 0.883562972 | |  | 0.00069716 |
| LPPR2 |  | darkmagenta |  | 0.952665511 | |  | 2.07E-05 |
| JUNB |  | darkmagenta |  | 0.882522959 | |  | 0.000721462 |
| IER2 |  | darkmagenta |  | 0.846387031 | |  | 0.002015126 |
| CD97 |  | darkmagenta |  | 0.850244692 | |  | 0.001829142 |
| EMR3 |  | darkmagenta |  | 0.956985158 | |  | 1.42E-05 |
| CYP4F3 |  | darkmagenta |  | 0.945789629 | |  | 3.54E-05 |
| EPS15L1 |  | darkmagenta |  | 0.949895288 | |  | 2.60E-05 |
| CHERP |  | darkmagenta |  | 0.926212513 | |  | 0.000118557 |
| MED26 |  | darkmagenta |  | 0.900136425 | |  | 0.000385113 |
| MYO9B |  | darkmagenta |  | 0.878779697 | |  | 0.000814074 |
| MAST3 |  | darkmagenta |  | 0.979965912 | |  | 6.88E-07 |
| ELL |  | darkmagenta |  | 0.881490855 | |  | 0.000746186 |
| LPAR2 |  | darkmagenta |  | 0.957909745 | |  | 1.30E-05 |
| GMIP |  | darkmagenta |  | 0.983720484 | |  | 3.01E-07 |
| KMT2B |  | darkmagenta |  | 0.914104478 | |  | 0.000214475 |
| RASGRP4 |  | darkmagenta |  | 0.931719747 | |  | 8.75E-05 |
| SAMD4B |  | darkmagenta |  | 0.949331599 | |  | 2.71E-05 |
| ZFP36 |  | darkmagenta |  | 0.803138087 | |  | 0.005142374 |
| SHKBP1 |  | darkmagenta |  | 0.934493017 | |  | 7.44E-05 |
| B3GNT8 |  | darkmagenta |  | 0.859813259 | |  | 0.00142171 |
| CEACAM3 |  | darkmagenta |  | 0.974519777 | |  | 1.79E-06 |
| DEDD2 |  | darkmagenta |  | 0.839435484 | |  | 0.002384221 |
| ERF |  | darkmagenta |  | 0.929651353 | |  | 9.84E-05 |
| CIC |  | darkmagenta |  | 0.938746699 | |  | 5.72E-05 |
| CEACAM1 |  | darkmagenta |  | 0.918209732 | |  | 0.000177218 |
| PLAUR |  | darkmagenta |  | 0.883900734 | |  | 0.000689397 |
| BCL3 |  | darkmagenta |  | 0.811518916 | |  | 0.004368017 |
| VASP |  | darkmagenta |  | 0.931989991 | |  | 8.62E-05 |
| PRKD2 |  | darkmagenta |  | 0.931217285 | |  | 9.01E-05 |
| C5AR1 |  | darkmagenta |  | 0.94843533 | |  | 2.91E-05 |
| C5AR2 |  | darkmagenta |  | 0.980893006 | |  | 5.70E-07 |
| DHX34 |  | darkmagenta |  | 0.982553639 | |  | 3.97E-07 |
| RCN3 |  | darkmagenta |  | 0.866375829 | |  | 0.001183377 |
| SIGLEC5 |  | darkmagenta |  | 0.82458319 | |  | 0.003332643 |
| FPR1 |  | darkmagenta |  | 0.935948493 | |  | 6.81E-05 |
| FPR2 |  | darkmagenta |  | 0.897955601 | |  | 0.000418733 |
| CNOT3 |  | darkmagenta |  | 0.822671022 | |  | 0.003471827 |
| MBOAT7 |  | darkmagenta |  | 0.951670261 | |  | 2.25E-05 |
| TSEN34 |  | darkmagenta |  | 0.811663183 | |  | 0.004355469 |
| LILRB3 |  | darkmagenta |  | 0.945312821 | |  | 3.66E-05 |
| LILRA6 |  | darkmagenta |  | 0.917520616 | |  | 0.000183111 |
| LILRB2 |  | darkmagenta |  | 0.83229386 | |  | 0.002811808 |
| FCAR |  | darkmagenta |  | 0.89878824 | |  | 0.000405653 |
| UBE2S |  | darkmagenta |  | 0.831589601 | |  | 0.002856765 |
| ZNF324 |  | darkmagenta |  | 0.823668212 | |  | 0.003398733 |
| CHMP2A |  | darkmagenta |  | 0.87836828 | |  | 0.000824755 |
| SLC12A7 |  | darkmagenta |  | -0.801452767 | |  | 0.005309161 |
| BASP1 |  | darkmagenta |  | 0.915794854 | |  | 0.000198499 |
| RICTOR |  | darkmagenta |  | 0.959349817 | |  | 1.14E-05 |
| CCDC125 |  | darkmagenta |  | 0.964778459 | |  | 6.45E-06 |
| ZFYVE16 |  | darkmagenta |  | 0.960957525 | |  | 9.70E-06 |
| CHD1 |  | darkmagenta |  | 0.898648341 | |  | 0.000407829 |
| LOC100289230 |  | darkmagenta |  | 0.818124076 | |  | 0.003819485 |
| PJA2 |  | darkmagenta |  | 0.837403642 | |  | 0.002500727 |
| FEM1C |  | darkmagenta |  | 0.815244117 | |  | 0.004052183 |
| LMNB1 |  | darkmagenta |  | 0.870022801 | |  | 0.001064261 |
| FNIP1 |  | darkmagenta |  | 0.964760569 | |  | 6.47E-06 |
| UBE2B |  | darkmagenta |  | 0.934588251 | |  | 7.40E-05 |
| FAM53C |  | darkmagenta |  | 0.867243398 | |  | 0.001154204 |
| CYSTM1 |  | darkmagenta |  | 0.836694616 | |  | 0.002542332 |
| SRA1 |  | darkmagenta |  | 0.819364738 | |  | 0.003722256 |
| ARAP3 |  | darkmagenta |  | 0.91265488 | |  | 0.000228913 |
| ARHGAP26 |  | darkmagenta |  | 0.978627024 | |  | 8.90E-07 |
| CCNJL |  | darkmagenta |  | 0.910646981 | |  | 0.000250075 |
| LCP2 |  | darkmagenta |  | 0.907751193 | |  | 0.000283084 |
| CPEB4 |  | darkmagenta |  | 0.8870751 | |  | 0.000619494 |
| RAB24 |  | darkmagenta |  | 0.926628965 | |  | 0.000115962 |
| MXD3 |  | darkmagenta |  | 0.948483088 | |  | 2.90E-05 |
| PDLIM7 |  | darkmagenta |  | 0.972929037 | |  | 2.27E-06 |
| DOK3 |  | darkmagenta |  | 0.883906068 | |  | 0.000689275 |
| BTNL8 |  | darkmagenta |  | 0.910807219 | |  | 0.000248336 |
| PITPNA |  | darkmagenta |  | 0.921662633 | |  | 0.000149772 |
| SLC43A2 |  | darkmagenta |  | 0.888284642 | |  | 0.000594274 |
| SCARF1 |  | darkmagenta |  | 0.86393203 | |  | 0.001268432 |
| RAP1GAP2 |  | darkmagenta |  | 0.853014445 | |  | 0.001703504 |
| CTNS |  | darkmagenta |  | -0.911324976 | |  | 0.000242775 |
| CAMKK1 |  | darkmagenta |  | 0.861085973 | |  | 0.001372987 |
| ARRB2 |  | darkmagenta |  | 0.973295224 | |  | 2.15E-06 |
| CXCL16 |  | darkmagenta |  | 0.955517556 | |  | 1.62E-05 |
| CHRNE |  | darkmagenta |  | 0.803285319 | |  | 0.005127983 |
| C17orf107 |  | darkmagenta |  | 0.922113022 | |  | 0.000146439 |
| RNASEK |  | darkmagenta |  | 0.823834229 | |  | 0.003386672 |
| ASGR2 |  | darkmagenta |  | -0.825513027 | |  | 0.003266431 |
| POLR2A |  | darkmagenta |  | 0.959163799 | |  | 1.16E-05 |
| KDM6B |  | darkmagenta |  | 0.896338281 | |  | 0.000445018 |
| VAMP2 |  | darkmagenta |  | 0.855330162 | |  | 0.00160336 |
| NDEL1 |  | darkmagenta |  | 0.969626434 | |  | 3.59E-06 |
| PIK3R5 |  | darkmagenta |  | 0.947326003 | |  | 3.16E-05 |
| MAP2K4 |  | darkmagenta |  | 0.812919051 | |  | 0.004247317 |
| FLOT2 |  | darkmagenta |  | 0.839178602 | |  | 0.00239873 |
| PHF12 |  | darkmagenta |  | 0.914252155 | |  | 0.000213043 |
| SSH2 |  | darkmagenta |  | 0.950726315 | |  | 2.43E-05 |
| CPD |  | darkmagenta |  | 0.884056182 | |  | 0.000685846 |
| EVI2B |  | darkmagenta |  | 0.929608367 | |  | 9.86E-05 |
| LOC440434 |  | darkmagenta |  | 0.834454757 | |  | 0.002677044 |
| CWC25 |  | darkmagenta |  | 0.850494378 | |  | 0.00181755 |
| LASP1 |  | darkmagenta |  | 0.921281636 | |  | 0.000152636 |
| MSL1 |  | darkmagenta |  | 0.939281704 | |  | 5.52E-05 |
| CASC3 |  | darkmagenta |  | 0.869552381 | |  | 0.001079113 |
| RARA |  | darkmagenta |  | 0.938234756 | |  | 5.91E-05 |
| STAT5B |  | darkmagenta |  | 0.874646122 | |  | 0.000926099 |
| STAT3 |  | darkmagenta |  | 0.915174795 | |  | 0.000204254 |
| FMNL1 |  | darkmagenta |  | 0.931159644 | |  | 9.04E-05 |
| PLEKHM1 |  | darkmagenta |  | 0.94779622 | |  | 3.05E-05 |
| NPEPPS |  | darkmagenta |  | 0.850365386 | |  | 0.001823532 |
| SPATA20 |  | darkmagenta |  | -0.900103501 | |  | 0.000385606 |
| SPAG9 |  | darkmagenta |  | 0.963789431 | |  | 7.20E-06 |
| TRIM25 |  | darkmagenta |  | 0.94704428 | |  | 3.23E-05 |
| VMP1 |  | darkmagenta |  | 0.929243812 | |  | 0.000100618 |
| CA4 |  | darkmagenta |  | 0.882328591 | |  | 0.000726072 |
| USP32 |  | darkmagenta |  | 0.957758669 | |  | 1.32E-05 |
| TANC2 |  | darkmagenta |  | 0.844750734 | |  | 0.002097999 |
| GNA13 |  | darkmagenta |  | 0.88806756 | |  | 0.000598744 |
| HN1 |  | darkmagenta |  | 0.87467828 | |  | 0.000925187 |
| UBALD2 |  | darkmagenta |  | 0.807874452 | |  | 0.004693679 |
| ST6GALNAC2 |  | darkmagenta |  | 0.971215673 | |  | 2.90E-06 |
| JMJD6 |  | darkmagenta |  | 0.987668131 | |  | 9.97E-08 |
| FLJ45079 |  | darkmagenta |  | 0.948142906 | |  | 2.97E-05 |
| LOC100653515 |  | darkmagenta |  | 0.897968922 | |  | 0.000418521 |
| CANT1 |  | darkmagenta |  | 0.965802382 | |  | 5.74E-06 |
| AATK |  | darkmagenta |  | 0.961706452 | |  | 8.98E-06 |
| ARHGDIA |  | darkmagenta |  | 0.806039482 | |  | 0.004864047 |
| SLC16A3 |  | darkmagenta |  | 0.903677636 | |  | 0.000334798 |
| CSNK1D |  | darkmagenta |  | 0.958648954 | |  | 1.22E-05 |
| SECTM1 |  | darkmagenta |  | 0.934310518 | |  | 7.52E-05 |
| B3GNTL1 |  | darkmagenta |  | 0.943518893 | |  | 4.16E-05 |
| METRNL |  | darkmagenta |  | 0.894167856 | |  | 0.000482168 |
| TOX4 |  | darkmagenta |  | 0.865803885 | |  | 0.0012029 |
| LRP10 |  | darkmagenta |  | 0.971686443 | |  | 2.72E-06 |
| PCK2 |  | darkmagenta |  | -0.897649743 | |  | 0.000423614 |
| NFKBIA |  | darkmagenta |  | 0.882785393 | |  | 0.000715273 |
| SOS2 |  | darkmagenta |  | 0.952225518 | |  | 2.15E-05 |
| PELI2 |  | darkmagenta |  | 0.860347831 | |  | 0.001401095 |
| ARID4A |  | darkmagenta |  | 0.807333712 | |  | 0.004743433 |
| HIF1A |  | darkmagenta |  | 0.830323299 | |  | 0.002938899 |
| KIAA0247 |  | darkmagenta |  | 0.977096912 | |  | 1.17E-06 |
| SIPA1L1 |  | darkmagenta |  | 0.952435757 | |  | 2.11E-05 |
| NUMB |  | darkmagenta |  | 0.928918798 | |  | 0.000102438 |
| AREL1 |  | darkmagenta |  | 0.925313682 | |  | 0.000124302 |
| C14orf159 |  | darkmagenta |  | 0.8609031 | |  | 0.001379912 |
| ITPK1 |  | darkmagenta |  | 0.871952566 | |  | 0.00100489 |
| SERPINA1 |  | darkmagenta |  | 0.846837538 | |  | 0.001992731 |
| CCNK |  | darkmagenta |  | 0.895916908 | |  | 0.000452061 |
| TECPR2 |  | darkmagenta |  | 0.883346608 | |  | 0.000702166 |
| EIF5 |  | darkmagenta |  | 0.824297574 | |  | 0.003353174 |
| NCOA1 |  | darkmagenta |  | 0.940548352 | |  | 5.09E-05 |
| NRBP1 |  | darkmagenta |  | 0.840638972 | |  | 0.002317088 |
| BRE-AS1 |  | darkmagenta |  | 0.959979236 | |  | 1.07E-05 |
| FOSL2 |  | darkmagenta |  | 0.895888587 | |  | 0.000452537 |
| PLB1 |  | darkmagenta |  | 0.895594688 | |  | 0.000457501 |
| YPEL5 |  | darkmagenta |  | 0.812824417 | |  | 0.004255399 |
| RMDN2 |  | darkmagenta |  | -0.874235528 | |  | 0.000937809 |
| DHX57 |  | darkmagenta |  | -0.873092924 | |  | 0.00097096 |
| RTN4 |  | darkmagenta |  | 0.815781738 | |  | 0.004007993 |
| RAB1A |  | darkmagenta |  | 0.972968913 | |  | 2.26E-06 |
| MXD1 |  | darkmagenta |  | 0.926714747 | |  | 0.000115433 |
| ASPRV1 |  | darkmagenta |  | 0.84958908 | |  | 0.001859836 |
| TGFA |  | darkmagenta |  | 0.878426901 | |  | 0.000823227 |
| DYSF |  | darkmagenta |  | 0.8975569 | |  | 0.000425104 |
| TMEM127 |  | darkmagenta |  | 0.899342346 | |  | 0.000397116 |
| RNF149 |  | darkmagenta |  | 0.951082442 | |  | 2.36E-05 |
| MAP4K4 |  | darkmagenta |  | 0.889829117 | |  | 0.000563173 |
| IL1R1 |  | darkmagenta |  | 0.944058132 | |  | 4.00E-05 |
| MAP3K2 |  | darkmagenta |  | 0.945598041 | |  | 3.59E-05 |
| ARHGAP15 |  | darkmagenta |  | 0.811249628 | |  | 0.00439151 |
| NMI |  | darkmagenta |  | 0.829916996 | |  | 0.002965608 |
| BAZ2B |  | darkmagenta |  | 0.969783797 | |  | 3.52E-06 |
| AHCTF1P1 |  | darkmagenta |  | 0.839313043 | |  | 0.002391128 |
| GCA |  | darkmagenta |  | 0.877586551 | |  | 0.000845333 |
| CIR1 |  | darkmagenta |  | 0.932634574 | |  | 8.30E-05 |
| NFE2L2 |  | darkmagenta |  | 0.941106318 | |  | 4.90E-05 |
| NABP1 |  | darkmagenta |  | 0.937705484 | |  | 6.11E-05 |
| CXCR2 |  | darkmagenta |  | 0.873769846 | |  | 0.000951219 |
| CXCR1 |  | darkmagenta |  | 0.870779128 | |  | 0.001040696 |
| ACSL3 |  | darkmagenta |  | 0.926206802 | |  | 0.000118592 |
| CUL3 |  | darkmagenta |  | 0.872886722 | |  | 0.000977033 |
| LRRFIP1 |  | darkmagenta |  | 0.822387025 | |  | 0.003492848 |
| RAF1 |  | darkmagenta |  | 0.920397837 | |  | 0.000159432 |
| IQSEC1 |  | darkmagenta |  | 0.963178179 | |  | 7.69E-06 |
| SLC6A6 |  | darkmagenta |  | 0.971283673 | |  | 2.87E-06 |
| RAB5A |  | darkmagenta |  | 0.893998585 | |  | 0.000485158 |
| CSRNP1 |  | darkmagenta |  | 0.8480727 | |  | 0.001932254 |
| NBEAL2 |  | darkmagenta |  | 0.948954485 | |  | 2.79E-05 |
| C3orf62 |  | darkmagenta |  | 0.877519833 | |  | 0.000847107 |
| USP4 |  | darkmagenta |  | 0.834726314 | |  | 0.002660444 |
| ARF4 |  | darkmagenta |  | 0.937766757 | |  | 6.08E-05 |
| EIF4E3 |  | darkmagenta |  | 0.908031233 | |  | 0.00027976 |
| LINC00877 |  | darkmagenta |  | 0.908286553 | |  | 0.000276754 |
| HSPBAP1 |  | darkmagenta |  | 0.863434039 | |  | 0.001286294 |
| ABTB1 |  | darkmagenta |  | 0.945223998 | |  | 3.69E-05 |
| RAB7A |  | darkmagenta |  | 0.835858716 | |  | 0.00259202 |
| TMCC1 |  | darkmagenta |  | 0.922428804 | |  | 0.000144135 |
| CEP63 |  | darkmagenta |  | 0.903513907 | |  | 0.000337011 |
| EPHB1 |  | darkmagenta |  | 0.942571071 | |  | 4.44E-05 |
| RNF13 |  | darkmagenta |  | 0.908233784 | |  | 0.000277373 |
| TSC22D2 |  | darkmagenta |  | 0.952797961 | |  | 2.05E-05 |
| MME |  | darkmagenta |  | 0.966999618 | |  | 4.99E-06 |
| CCNL1 |  | darkmagenta |  | 0.867704941 | |  | 0.001138899 |
| FNDC3B |  | darkmagenta |  | 0.968248349 | |  | 4.28E-06 |
| ATP11B |  | darkmagenta |  | 0.871843114 | |  | 0.001008191 |
| B3GNT5 |  | darkmagenta |  | 0.950377255 | |  | 2.50E-05 |
| VPS8 |  | darkmagenta |  | 0.900486279 | |  | 0.00037991 |
| IL1RAP |  | darkmagenta |  | 0.884104385 | |  | 0.000684748 |
| ATP13A3 |  | darkmagenta |  | 0.81812481 | |  | 0.003819427 |
| PAK2 |  | darkmagenta |  | 0.85125776 | |  | 0.001782437 |
| FAM157A |  | darkmagenta |  | 0.885821838 | |  | 0.000646441 |
| LINC01001 |  | darkmagenta |  | 0.890115635 | |  | 0.000557537 |
| IFITM2 |  | darkmagenta |  | 0.971794415 | |  | 2.68E-06 |
| LSP1 |  | darkmagenta |  | 0.871086119 | |  | 0.00103124 |
| NUP98 |  | darkmagenta |  | 0.931938795 | |  | 8.64E-05 |
| RHOG |  | darkmagenta |  | 0.985833334 | |  | 1.73E-07 |
| DENND5A |  | darkmagenta |  | 0.956193526 | |  | 1.53E-05 |
| TMEM41B |  | darkmagenta |  | 0.854976565 | |  | 0.001618368 |
| RNF141 |  | darkmagenta |  | 0.825972712 | |  | 0.00323405 |
| MRVI1 |  | darkmagenta |  | 0.892607929 | |  | 0.000510236 |
| ARNTL |  | darkmagenta |  | 0.922600637 | |  | 0.000142892 |
| PRRG4 |  | darkmagenta |  | 0.875365956 | |  | 0.000905829 |
| CD82 |  | darkmagenta |  | 0.930560764 | |  | 9.35E-05 |
| TP53I11 |  | darkmagenta |  | 0.855667121 | |  | 0.001589152 |
| PHF21A |  | darkmagenta |  | 0.941423 | |  | 4.80E-05 |
| SPI1 |  | darkmagenta |  | 0.958050672 | |  | 1.29E-05 |
| STX3 |  | darkmagenta |  | 0.96681024 | |  | 5.10E-06 |
| SLC15A3 |  | darkmagenta |  | 0.905592049 | |  | 0.000309701 |
| BEST1 |  | darkmagenta |  | 0.959300416 | |  | 1.14E-05 |
| PYGM |  | darkmagenta |  | 0.861598523 | |  | 0.001353712 |
| ATG2A |  | darkmagenta |  | 0.927764828 | |  | 0.000109099 |
| NEAT1 |  | darkmagenta |  | 0.922190404 | |  | 0.000145872 |
| MIR612 |  | darkmagenta |  | 0.933271371 | |  | 8.00E-05 |
| MALAT1 |  | darkmagenta |  | 0.886755643 | |  | 0.000626283 |
| KCNK7 |  | darkmagenta |  | 0.883550782 | |  | 0.000697441 |
| MAP3K11 |  | darkmagenta |  | 0.868370112 | |  | 0.001117103 |
| AP5B1 |  | darkmagenta |  | 0.973738694 | |  | 2.02E-06 |
| PDE2A |  | darkmagenta |  | 0.897402157 | |  | 0.000427596 |
| ARAP1 |  | darkmagenta |  | 0.959734276 | |  | 1.10E-05 |
| ATG16L2 |  | darkmagenta |  | 0.922058355 | |  | 0.000146841 |
| DGAT2 |  | darkmagenta |  | 0.937318408 | |  | 6.26E-05 |
| PICALM |  | darkmagenta |  | 0.915092423 | |  | 0.000205028 |
| SORL1 |  | darkmagenta |  | 0.964677728 | |  | 6.53E-06 |
| ADIPOR2 |  | darkmagenta |  | -0.817687013 | |  | 0.003854167 |
| CHD4 |  | darkmagenta |  | 0.930241882 | |  | 9.52E-05 |
| MLF2 |  | darkmagenta |  | 0.900910163 | |  | 0.000373676 |
| C1RL |  | darkmagenta |  | 0.862439679 | |  | 0.001322506 |
| C1RL-AS1 |  | darkmagenta |  | 0.829024092 | |  | 0.003024918 |
| GABARAPL1 |  | darkmagenta |  | 0.903383258 | |  | 0.000338785 |
| MANSC1 |  | darkmagenta |  | 0.914939534 | |  | 0.000206469 |
| KIAA1551 |  | darkmagenta |  | 0.863638427 | |  | 0.001278941 |
| LRRK2 |  | darkmagenta |  | 0.913378533 | |  | 0.000221619 |
| VDR |  | darkmagenta |  | 0.947234109 | |  | 3.18E-05 |
| KMT2D |  | darkmagenta |  | 0.915410789 | |  | 0.000202049 |
| DIP2B |  | darkmagenta |  | 0.912689524 | |  | 0.000228559 |
| ARHGAP9 |  | darkmagenta |  | 0.833222126 | |  | 0.002753333 |
| DDIT3 |  | darkmagenta |  | 0.834729933 | |  | 0.002660223 |
| RAB21 |  | darkmagenta |  | 0.912754106 | |  | 0.000227902 |
| OSBPL8 |  | darkmagenta |  | 0.84781964 | |  | 0.001944535 |
| PLXNC1 |  | darkmagenta |  | 0.953015397 | |  | 2.01E-05 |
| TMCC3 |  | darkmagenta |  | 0.973345278 | |  | 2.14E-06 |
| HAL |  | darkmagenta |  | 0.865486736 | |  | 0.001213825 |
| UHRF1BP1L |  | darkmagenta |  | 0.885074957 | |  | 0.000662902 |
| CHST11 |  | darkmagenta |  | 0.909715306 | |  | 0.000260368 |
| MED13L |  | darkmagenta |  | 0.902529891 | |  | 0.000350542 |
| LINC00173 |  | darkmagenta |  | 0.943875648 | |  | 4.06E-05 |
| MAP1LC3B2 |  | darkmagenta |  | 0.836634756 | |  | 0.002545867 |
| SUDS3 |  | darkmagenta |  | 0.875255232 | |  | 0.000908926 |
| PXN |  | darkmagenta |  | 0.957620224 | |  | 1.34E-05 |
| HCAR2 |  | darkmagenta |  | 0.835051081 | |  | 0.002640689 |
| GLT1D1 |  | darkmagenta |  | 0.948842691 | |  | 2.82E-05 |
| ULK1 |  | darkmagenta |  | 0.886038852 | |  | 0.000641715 |
| S100P |  | darkmagenta |  | 0.903466703 | |  | 0.000337652 |
| KIAA0232 |  | darkmagenta |  | 0.986093836 | |  | 1.61E-07 |
| BOD1L1 |  | darkmagenta |  | 0.962654242 | |  | 8.13E-06 |
| CPEB2 |  | darkmagenta |  | 0.915688884 | |  | 0.000199474 |
| RBPJ |  | darkmagenta |  | 0.855116745 | |  | 0.001612406 |
| TLR1 |  | darkmagenta |  | 0.990433625 | |  | 3.62E-08 |
| RBM47 |  | darkmagenta |  | 0.937575252 | |  | 6.16E-05 |
| NSUN7 |  | darkmagenta |  | 0.957094123 | |  | 1.41E-05 |
| MOB1B |  | darkmagenta |  | -0.861137191 | |  | 0.001371052 |
| CXCL1 |  | darkmagenta |  | 0.837926369 | |  | 0.00247037 |
| CCNG2 |  | darkmagenta |  | 0.804208938 | |  | 0.00503836 |
| THAP9-AS1 |  | darkmagenta |  | 0.80696391 | |  | 0.004777676 |
| WDFY3 |  | darkmagenta |  | 0.936591953 | |  | 6.55E-05 |
| HERC3 |  | darkmagenta |  | 0.903851527 | |  | 0.000332459 |
| FAM13A-AS1 |  | darkmagenta |  | 0.922597547 | |  | 0.000142914 |
| PPP3CA |  | darkmagenta |  | 0.885730163 | |  | 0.000648445 |
| TET2 |  | darkmagenta |  | 0.937394689 | |  | 6.23E-05 |
| ALPK1 |  | darkmagenta |  | 0.976880823 | |  | 1.22E-06 |
| TMEM154 |  | darkmagenta |  | 0.922179201 | |  | 0.000145954 |
| RAPGEF2 |  | darkmagenta |  | 0.921079567 | |  | 0.00015417 |
| DDX60L |  | darkmagenta |  | 0.881261941 | |  | 0.000751752 |
| ING2 |  | darkmagenta |  | 0.903539912 | |  | 0.000336659 |
| PFKFB3 |  | darkmagenta |  | 0.877116503 | |  | 0.000857885 |
| RP11-563J2.2 |  | darkmagenta |  | 0.850648338 | |  | 0.001810428 |
| APBB1IP |  | darkmagenta |  | 0.906556121 | |  | 0.000297599 |
| ZNF438 |  | darkmagenta |  | 0.913472184 | |  | 0.000220688 |
| IPMK |  | darkmagenta |  | 0.892857311 | |  | 0.000505671 |
| NRBF2 |  | darkmagenta |  | 0.921964137 | |  | 0.000147535 |
| JMJD1C |  | darkmagenta |  | 0.825362664 | |  | 0.003277074 |
| SRGN |  | darkmagenta |  | 0.930238432 | |  | 9.52E-05 |
| ZSWIM8 |  | darkmagenta |  | 0.921260401 | |  | 0.000152796 |
| FAS |  | darkmagenta |  | 0.889902518 | |  | 0.000561725 |
| NFKB2 |  | darkmagenta |  | 0.803670043 | |  | 0.005090514 |
| ITPRIP |  | darkmagenta |  | 0.919110214 | |  | 0.000169731 |
| SHOC2 |  | darkmagenta |  | 0.818763842 | |  | 0.003769123 |
| PDZD8 |  | darkmagenta |  | 0.943087727 | |  | 4.28E-05 |
| CACUL1 |  | darkmagenta |  | 0.899955789 | |  | 0.00038782 |
| PTPRE |  | darkmagenta |  | 0.899760556 | |  | 0.000390761 |
| ADAM8 |  | darkmagenta |  | 0.950629145 | |  | 2.45E-05 |
| GCNT2 |  | darkmagenta |  | -0.87341302 | |  | 0.000961588 |
| NEDD9 |  | darkmagenta |  | 0.933253496 | |  | 8.01E-05 |
| PHACTR1 |  | darkmagenta |  | 0.807674419 | |  | 0.00471204 |
| TBC1D7 |  | darkmagenta |  | 0.805075733 | |  | 0.004955274 |
| FAM8A1 |  | darkmagenta |  | 0.966777575 | |  | 5.12E-06 |
| TDP2 |  | darkmagenta |  | 0.938316326 | |  | 5.88E-05 |
| PPP1R10 |  | darkmagenta |  | 0.904671486 | |  | 0.00032159 |
| PPP1R18 |  | darkmagenta |  | 0.895646102 | |  | 0.00045663 |
| FLOT1 |  | darkmagenta |  | 0.936566994 | |  | 6.56E-05 |
| IER3 |  | darkmagenta |  | 0.828402831 | |  | 0.003066685 |
| LST1 |  | darkmagenta |  | 0.811355963 | |  | 0.004382223 |
| DDAH2 |  | darkmagenta |  | 0.899364944 | |  | 0.000396771 |
| AGER |  | darkmagenta |  | 0.822369052 | |  | 0.003494182 |
| PBX2 |  | darkmagenta |  | 0.951607961 | |  | 2.26E-05 |
| GPSM3 |  | darkmagenta |  | 0.82512327 | |  | 0.003294069 |
| BRD2 |  | darkmagenta |  | 0.802387733 | |  | 0.005216164 |
| SRPK1 |  | darkmagenta |  | 0.958550534 | |  | 1.23E-05 |
| MAPK13 |  | darkmagenta |  | 0.832995927 | |  | 0.002767501 |
| CDKN1A |  | darkmagenta |  | -0.840488156 | |  | 0.002325425 |
| TREML2 |  | darkmagenta |  | 0.902831574 | |  | 0.000346352 |
| TREM1 |  | darkmagenta |  | 0.965835106 | |  | 5.72E-06 |
| UBR2 |  | darkmagenta |  | 0.891500992 | |  | 0.000530866 |
| NFKBIE |  | darkmagenta |  | 0.882270241 | |  | 0.00072746 |
| ELOVL5 |  | darkmagenta |  | 0.89391999 | |  | 0.000486551 |
| RPS16P5 |  | darkmagenta |  | 0.903303577 | |  | 0.000339871 |
| PTP4A1 |  | darkmagenta |  | 0.853482303 | |  | 0.001682916 |
| CYB5R4 |  | darkmagenta |  | 0.839080587 | |  | 0.002404282 |
| ZNF292 |  | darkmagenta |  | 0.821217094 | |  | 0.003580412 |
| PNRC1 |  | darkmagenta |  | 0.818233899 | |  | 0.003810806 |
| CDK19 |  | darkmagenta |  | 0.914324754 | |  | 0.000212342 |
| MARCKS |  | darkmagenta |  | 0.827965971 | |  | 0.003096302 |
| VNN2 |  | darkmagenta |  | 0.852057702 | |  | 0.001746172 |
| SOD2 |  | darkmagenta |  | 0.934410567 | |  | 7.48E-05 |
| IGF2R |  | darkmagenta |  | 0.961710047 | |  | 8.98E-06 |
| LOC729603 |  | darkmagenta |  | 0.948660611 | |  | 2.86E-05 |
| B4GALT1 |  | darkmagenta |  | 0.902289936 | |  | 0.000353901 |
| UBE2R2 |  | darkmagenta |  | 0.928951038 | |  | 0.000102257 |
| CCIN |  | darkmagenta |  | 0.880397984 | |  | 0.000773033 |
| CNTNAP3B |  | darkmagenta |  | 0.847676195 | |  | 0.001951521 |
| TLE4 |  | darkmagenta |  | 0.875926855 | |  | 0.00089026 |
| NFIL3 |  | darkmagenta |  | 0.941599074 | |  | 4.74E-05 |
| NINJ1 |  | darkmagenta |  | 0.843372567 | |  | 0.002169686 |
| ABCA1 |  | darkmagenta |  | 0.802778187 | |  | 0.005177673 |
| DNAJC25-GNG10 |  | darkmagenta |  | 0.920884863 | |  | 0.00015566 |
| GNG10 |  | darkmagenta |  | 0.926101114 | |  | 0.000119258 |
| HSDL2 |  | darkmagenta |  | 0.874968719 | |  | 0.000916975 |
| NR6A1 |  | darkmagenta |  | 0.976300715 | |  | 1.34E-06 |
| GOLGA2 |  | darkmagenta |  | 0.872887258 | |  | 0.000977017 |
| NTNG2 |  | darkmagenta |  | 0.910808428 | |  | 0.000248322 |
| NOTCH1 |  | darkmagenta |  | 0.983201496 | |  | 3.41E-07 |
| PNPLA7 |  | darkmagenta |  | -0.809060912 | |  | 0.004585818 |
| FAM157B |  | darkmagenta |  | 0.875287229 | |  | 0.00090803 |
| TNRC18 |  | darkmagenta |  | 0.905396792 | |  | 0.000312195 |
| CREB5 |  | darkmagenta |  | 0.89003877 | |  | 0.000559045 |
| EEPD1 |  | darkmagenta |  | 0.884563895 | |  | 0.00067434 |
| PURB |  | darkmagenta |  | 0.826769052 | |  | 0.0031785 |
| RABGEF1 |  | darkmagenta |  | 0.913043845 | |  | 0.000224971 |
| DTX2 |  | darkmagenta |  | 0.834310078 | |  | 0.002685919 |
| STEAP4 |  | darkmagenta |  | 0.975753664 | |  | 1.47E-06 |
| CDK14 |  | darkmagenta |  | 0.80989954 | |  | 0.004510656 |
| LMTK2 |  | darkmagenta |  | 0.918378811 | |  | 0.000175794 |
| PILRA |  | darkmagenta |  | 0.903041818 | |  | 0.000343454 |
| TSC22D4 |  | darkmagenta |  | 0.97728761 | |  | 1.13E-06 |
| GNB2 |  | darkmagenta |  | 0.938917895 | |  | 5.66E-05 |
| EPHB4 |  | darkmagenta |  | 0.854915593 | |  | 0.001620966 |
| SLC12A9 |  | darkmagenta |  | 0.865603941 | |  | 0.001209779 |
| FBXL13 |  | darkmagenta |  | 0.91384093 | |  | 0.000217049 |
| KMT2E-AS1 |  | darkmagenta |  | 0.853312001 | |  | 0.001690389 |
| NAMPT |  | darkmagenta |  | 0.952361422 | |  | 2.13E-05 |
| CCDC71L |  | darkmagenta |  | 0.841315108 | |  | 0.002279977 |
| PNPLA8 |  | darkmagenta |  | 0.862973525 | |  | 0.001302974 |
| DOCK4 |  | darkmagenta |  | 0.861086299 | |  | 0.001372975 |
| IFRD1 |  | darkmagenta |  | 0.884723097 | |  | 0.000670762 |
| LRRC4 |  | darkmagenta |  | 0.941146955 | |  | 4.89E-05 |
| LINC01000 |  | darkmagenta |  | 0.816675722 | |  | 0.003935278 |
| AC058791.1 |  | darkmagenta |  | 0.897866344 | |  | 0.000420153 |
| LINC-PINT |  | darkmagenta |  | 0.886142836 | |  | 0.000639459 |
| MGAM |  | darkmagenta |  | 0.935325177 | |  | 7.08E-05 |
| ZNF746 |  | darkmagenta |  | 0.874568961 | |  | 0.000928292 |
| ZNF467 |  | darkmagenta |  | 0.896264004 | |  | 0.000446254 |
| KMT2C |  | darkmagenta |  | 0.907837769 | |  | 0.000282053 |
| ERI1 |  | darkmagenta |  | 0.800562223 | |  | 0.005398834 |
| PPP1R3B |  | darkmagenta |  | 0.958749862 | |  | 1.21E-05 |
| BIN3 |  | darkmagenta |  | 0.920382112 | |  | 0.000159555 |
| BIN3-IT1 |  | darkmagenta |  | 0.810591792 | |  | 0.004449279 |
| LOC254896 |  | darkmagenta |  | 0.965711319 | |  | 5.80E-06 |
| TNFRSF10C |  | darkmagenta |  | 0.95484512 | |  | 1.72E-05 |
| DOCK5 |  | darkmagenta |  | 0.984152783 | |  | 2.71E-07 |
| PPP2R2A |  | darkmagenta |  | 0.854294596 | |  | 0.0016476 |
| POLB |  | darkmagenta |  | 0.831327912 | |  | 0.002873602 |
| SPIDR |  | darkmagenta |  | -0.875391558 | |  | 0.000905114 |
| RB1CC1 |  | darkmagenta |  | 0.850773961 | |  | 0.001804633 |
| YTHDF3 |  | darkmagenta |  | 0.923406397 | |  | 0.000137171 |
| LY96 |  | darkmagenta |  | 0.96829439 | |  | 4.26E-06 |
| NBN |  | darkmagenta |  | 0.835432653 | |  | 0.002617614 |
| CPQ |  | darkmagenta |  | 0.915615271 | |  | 0.000200153 |
| LAPTM4B |  | darkmagenta |  | -0.807077701 | |  | 0.00476712 |
| AZIN1 |  | darkmagenta |  | 0.876762641 | |  | 0.000867424 |
| FAM49B |  | darkmagenta |  | 0.955373703 | |  | 1.64E-05 |
| TMEM71 |  | darkmagenta |  | 0.840912077 | |  | 0.002302046 |
| SLA |  | darkmagenta |  | 0.814076775 | |  | 0.004149332 |
| DENND3 |  | darkmagenta |  | 0.903977131 | |  | 0.000330776 |
| SLC45A4 |  | darkmagenta |  | 0.924252411 | |  | 0.000131347 |
| ZC3H3 |  | darkmagenta |  | 0.915101036 | |  | 0.000204947 |
| OPLAH |  | darkmagenta |  | 0.856307897 | |  | 0.001562386 |
| TBL1X |  | darkmagenta |  | 0.94467278 | |  | 3.83E-05 |
| WWC3 |  | darkmagenta |  | 0.947080749 | |  | 3.22E-05 |
| SYAP1 |  | darkmagenta |  | 0.911696305 | |  | 0.000238844 |
| GK |  | darkmagenta |  | 0.919453047 | |  | 0.000166942 |
| USP9X |  | darkmagenta |  | 0.847858809 | |  | 0.00194263 |
| CDK16 |  | darkmagenta |  | 0.839231821 | |  | 0.002395719 |
| WAS |  | darkmagenta |  | 0.926363376 | |  | 0.000117612 |
| SLC35A2 |  | darkmagenta |  | 0.827462059 | |  | 0.003130719 |
| TFE3 |  | darkmagenta |  | 0.930074374 | |  | 9.61E-05 |
| MED12 |  | darkmagenta |  | 0.963976548 | |  | 7.05E-06 |
| NLGN3 |  | darkmagenta |  | 0.929456653 | |  | 9.94E-05 |
| NHSL2 |  | darkmagenta |  | 0.810615078 | |  | 0.004447225 |
| BRWD3 |  | darkmagenta |  | 0.904331304 | |  | 0.000326067 |
| LAMP2 |  | darkmagenta |  | 0.854709666 | |  | 0.001629763 |
| AFF2 |  | darkmagenta |  | 0.928735096 | |  | 0.000103478 |
| IDS |  | darkmagenta |  | 0.926341536 | |  | 0.000117748 |
| LATS2 |  | darkmagenta |  | 0.953519803 | |  | 1.93E-05 |
| ALOX5AP |  | darkmagenta |  | 0.891488178 | |  | 0.000531108 |
| KIAA0226L |  | darkmagenta |  | 0.817669233 | |  | 0.003855582 |
| ITM2B |  | darkmagenta |  | 0.865172166 | |  | 0.001224734 |
| INTS6 |  | darkmagenta |  | 0.863998099 | |  | 0.001266076 |
| DHRS12 |  | darkmagenta |  | 0.820151104 | |  | 0.003661557 |
| TNFSF13B |  | darkmagenta |  | 0.84966028 | |  | 0.001856485 |
| BID |  | darkmagenta |  | 0.931511341 | |  | 8.86E-05 |
| LINC00528 |  | darkmagenta |  | 0.813118953 | |  | 0.004230281 |
| SPECC1L-ADORA2A |  | darkmagenta |  | 0.80966901 | |  | 0.004531228 |
| ADORA2A |  | darkmagenta |  | 0.839470442 | |  | 0.002382251 |
| ADORA2A-AS1 |  | darkmagenta |  | 0.856462902 | |  | 0.001555961 |
| UPB1 |  | darkmagenta |  | 0.876361708 | |  | 0.000878325 |
| THOC5 |  | darkmagenta |  | 0.867125557 | |  | 0.001158136 |
| LIMK2 |  | darkmagenta |  | 0.844662111 | |  | 0.002102557 |
| NCF4 |  | darkmagenta |  | 0.96449792 | |  | 6.66E-06 |
| CSF2RB |  | darkmagenta |  | 0.870162982 | |  | 0.001059864 |
| RAC2 |  | darkmagenta |  | 0.82793406 | |  | 0.003098473 |
| TMEM184B |  | darkmagenta |  | 0.955144825 | |  | 1.68E-05 |
| GTPBP1 |  | darkmagenta |  | 0.898798596 | |  | 0.000405492 |
| APOBEC3A |  | darkmagenta |  | 0.856637935 | |  | 0.001548729 |
| APOBEC3B |  | darkmagenta |  | 0.947618061 | |  | 3.09E-05 |
| NFAM1 |  | darkmagenta |  | 0.861949012 | |  | 0.001340645 |
| ARFGAP3 |  | darkmagenta |  | 0.834116202 | |  | 0.002697844 |
| PACSIN2 |  | darkmagenta |  | 0.81534211 | |  | 0.004044102 |
| PANX2 |  | darkmagenta |  | 0.974375112 | |  | 1.83E-06 |
| SLC12A6 |  | darkmagenta |  | 0.982458346 | |  | 4.06E-07 |
| NOP10 |  | darkmagenta |  | 0.821410581 | |  | 0.003565823 |
| AQP9 |  | darkmagenta |  | 0.96414151 | |  | 6.93E-06 |
| RAB8B |  | darkmagenta |  | 0.922789609 | |  | 0.000141535 |
| DAPK2 |  | darkmagenta |  | 0.891529558 | |  | 0.000530326 |
| OAZ2 |  | darkmagenta |  | 0.908943549 | |  | 0.000269128 |
| ANKDD1A |  | darkmagenta |  | 0.825890741 | |  | 0.003239807 |
| TLE3 |  | darkmagenta |  | 0.932038999 | |  | 8.59E-05 |
| PPCDC |  | darkmagenta |  | 0.855129513 | |  | 0.001611864 |
| C15orf39 |  | darkmagenta |  | 0.887763028 | |  | 0.000605056 |
| MTHFS |  | darkmagenta |  | 0.872246534 | |  | 0.000996062 |
| ST20-MTHFS |  | darkmagenta |  | 0.874311149 | |  | 0.000935644 |
| ST20 |  | darkmagenta |  | 0.914983499 | |  | 0.000206054 |
| C15orf37 |  | darkmagenta |  | 0.816311808 | |  | 0.003964763 |
| BCL2A1 |  | darkmagenta |  | 0.909024708 | |  | 0.000268197 |
| ABHD2 |  | darkmagenta |  | 0.908916641 | |  | 0.000269437 |
| ANPEP |  | darkmagenta |  | 0.895324987 | |  | 0.000462091 |
| MCTP2 |  | darkmagenta |  | 0.938355887 | |  | 5.86E-05 |
| IGF1R |  | darkmagenta |  | 0.956887149 | |  | 1.43E-05 |
| CHSY1 |  | darkmagenta |  | 0.920463056 | |  | 0.000158923 |
| CBX3P2 |  | darkmagenta |  | 0.858868297 | |  | 0.001458689 |
| SMCHD1 |  | darkmagenta |  | 0.948848017 | |  | 2.82E-05 |
| PPP4R1 |  | darkmagenta |  | 0.942053922 | |  | 4.60E-05 |
| CTIF |  | darkmagenta |  | -0.878743613 | |  | 0.000815006 |
| CTDP1 |  | darkmagenta |  | 0.816961928 | |  | 0.0039122 |
| SIRPB1 |  | darkmagenta |  | 0.86095464 | |  | 0.001377958 |
| SIRPA |  | darkmagenta |  | 0.853282717 | |  | 0.001691677 |
| RNF24 |  | darkmagenta |  | 0.924159999 | |  | 0.000131974 |
| RASSF2 |  | darkmagenta |  | 0.898351168 | |  | 0.000412481 |
| GPCPD1 |  | darkmagenta |  | 0.873190224 | |  | 0.000968104 |
| RALGAPA2 |  | darkmagenta |  | 0.973490205 | |  | 2.09E-06 |
| GZF1 |  | darkmagenta |  | 0.824642959 | |  | 0.003328359 |
| HCK |  | darkmagenta |  | 0.806684062 | |  | 0.004803706 |
| MAP1LC3A |  | darkmagenta |  | 0.808465053 | |  | 0.004639763 |
| SOGA1 |  | darkmagenta |  | -0.867300264 | |  | 0.001152311 |
| SLPI |  | darkmagenta |  | 0.827315901 | |  | 0.003140753 |
| ZNFX1 |  | darkmagenta |  | 0.929844146 | |  | 9.73E-05 |
| B4GALT5 |  | darkmagenta |  | 0.851342623 | |  | 0.001778564 |
| CEBPB |  | darkmagenta |  | 0.876033812 | |  | 0.000887314 |
| RP11-290F20.3 |  | darkmagenta |  | 0.987195511 | |  | 1.16E-07 |
| SUMO1P1 |  | darkmagenta |  | 0.928922135 | |  | 0.00010242 |
| RTFDC1 |  | darkmagenta |  | 0.807655751 | |  | 0.004713757 |
| FAM209A |  | darkmagenta |  | 0.929522337 | |  | 9.91E-05 |
| PPP4R1L |  | darkmagenta |  | 0.927300904 | |  | 0.000111865 |
| STX16 |  | darkmagenta |  | 0.875587535 | |  | 0.000899655 |
| STX16-NPEPL1 |  | darkmagenta |  | 0.882550819 | |  | 0.000720803 |
| OSBPL2 |  | darkmagenta |  | 0.977751483 | |  | 1.04E-06 |
| SYNJ1 |  | darkmagenta |  | 0.964058179 | |  | 6.99E-06 |
| IFNAR2 |  | darkmagenta |  | 0.91729878 | |  | 0.000185038 |
| IFNAR1 |  | darkmagenta |  | 0.923007601 | |  | 0.000139981 |
| ETS2 |  | darkmagenta |  | 0.906843776 | |  | 0.000294056 |
| U2AF1 |  | darkmagenta |  | 0.892048134 | |  | 0.000520594 |
| AGPAT3 |  | darkmagenta |  | -0.896877833 | |  | 0.000436119 |
| POFUT2 |  | darkmagenta |  | 0.92508008 | |  | 0.000125828 |
| SLC19A1 |  | darkmagenta |  | 0.976004301 | |  | 1.41E-06 |
