# Supplementary material for: Integrated multi-omics analysis identifies candidate eRNA-associated signatures shared between osteoarthritis and type 2 diabetes
Source: Front Genet. 2026 Jul 17;17:1875546. doi: 10.3389/fgene.2026.1875546 (PMC13423288; doi:10.3389/fgene.2026.1875546)
Supplement: Supplementary file 2 [file Supplementaryfile1.pdf]

Supplementary Figures

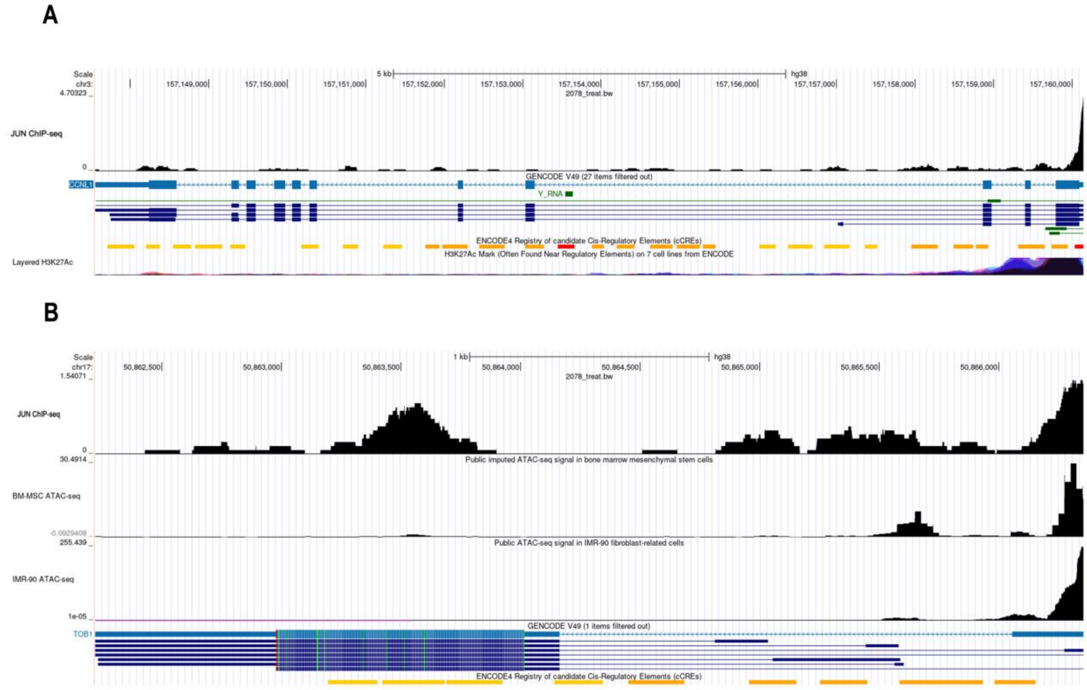

Supplementary Figure 1. ChIP-seq and ATAC-seq analysis of the predicted eRNA-related regulatory network.

(A) Public JUN ChIP-seq signals around the CCNL1 locus.
(B) Public JUN ChIP-seq signals around the TOB1-related regulatory region, together with imputed ATAC-seq signals from bone marrow mesenchymal stem cells, experimental ATAC-seq signals from IMR-90 fibroblast-related cells, and ENCODE candidate cis-regulatory element (cCRE) annotations.

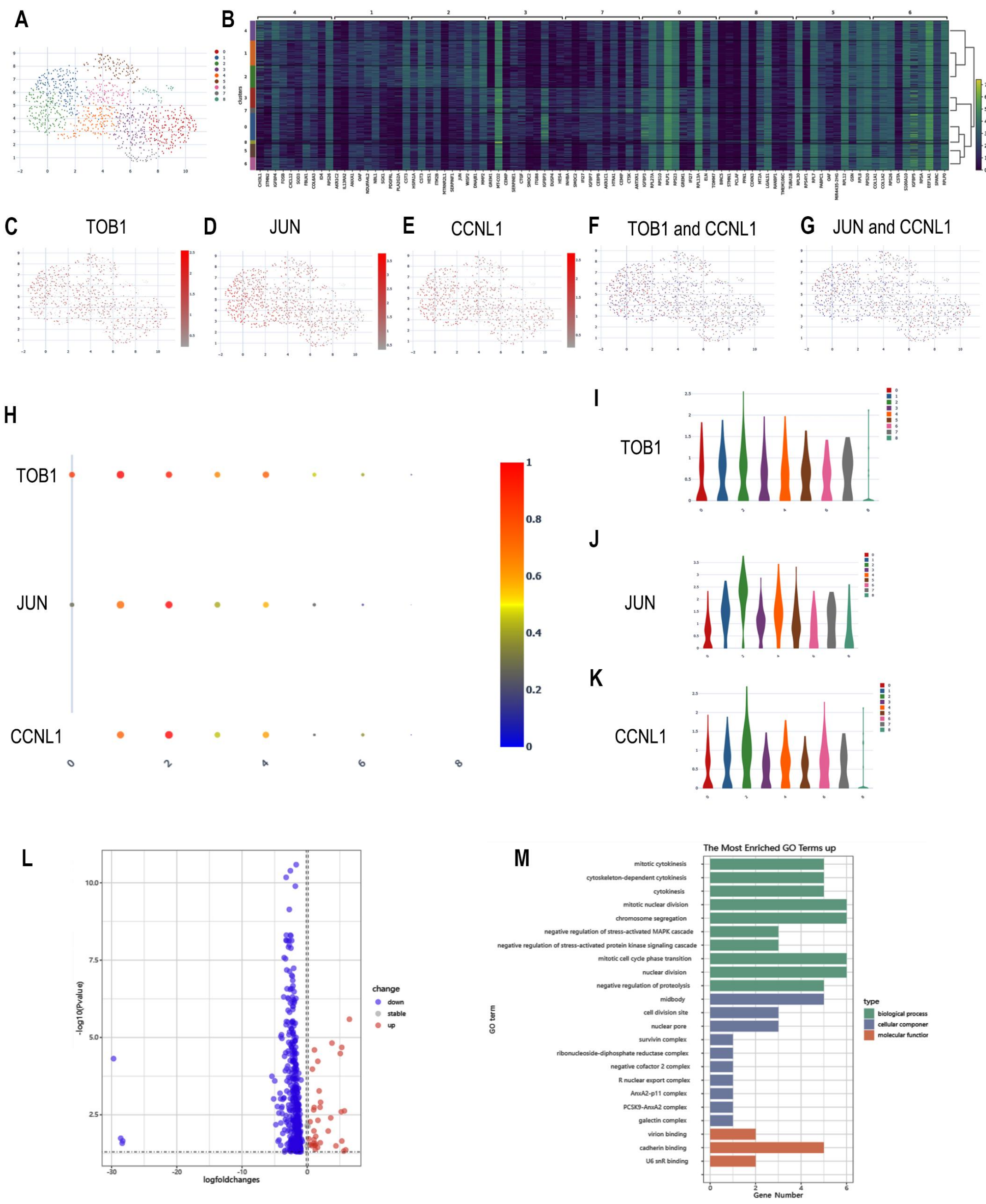

Supplementary Figure 2. Single-cell RNA sequencing validation of TOB1, JUN, and CCNL1 expression.

(A) t-SNE plot showing nine distinct fibroblast clusters in OA synovium. (B) Heatmap of top marker genes for each cluster. (C–E) Feature plots illustrating spatial expression of TOB1, JUN, and CCNL1. (F–G) Co-expression plots demonstrating overlapping TOB1–CCNL1 and JUN–CCNL1 expression. (H–K) Dot and violin plots showing differential expression of these genes across clusters. (L–M) Volcano plot and GO enrichment analysis highlighting the transcriptional program of Cluster 8.

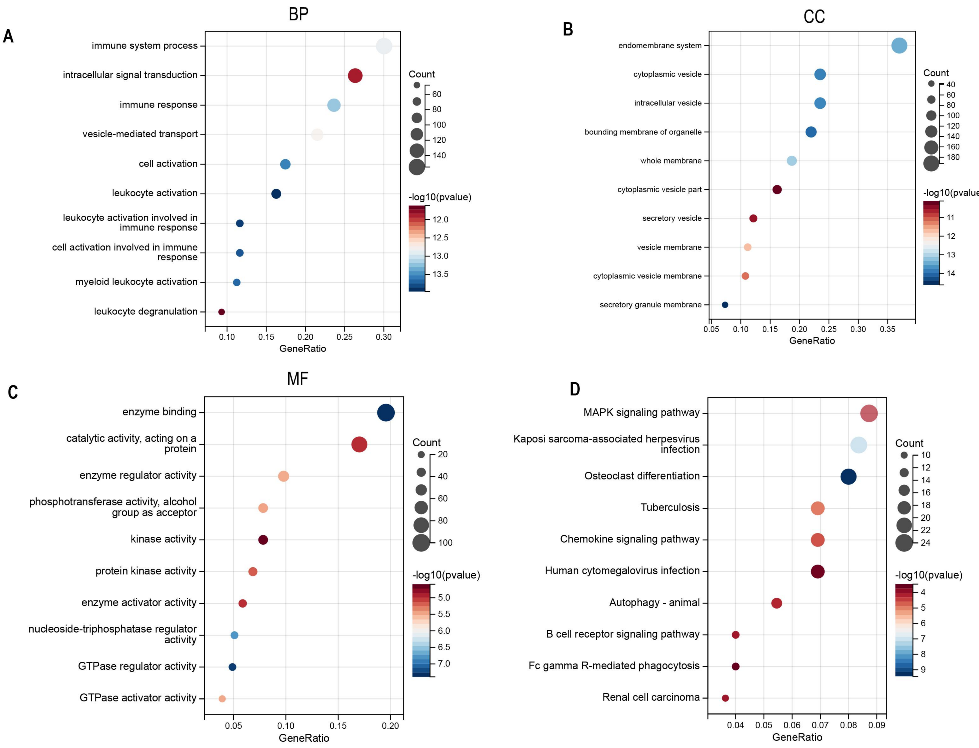

Supplementary Figure 3. Functional enrichment analysis of hub genes in the darkmagenta module.

A total of 611 hub genes identified in the darkmagenta module were subjected to GO and KEGG enrichment analyses. (A–C) GO enrichment analysis showing the top enriched terms in biological process (BP), cellular component (CC), and molecular function (MF), respectively. (D) KEGG pathway enrichment analysis of the hub genes, highlighting enrichment in MAPK signaling-related pathways. The x-axis represents GeneRatio, dot size indicates the number of enriched genes, and dot color represents −log10(P value).

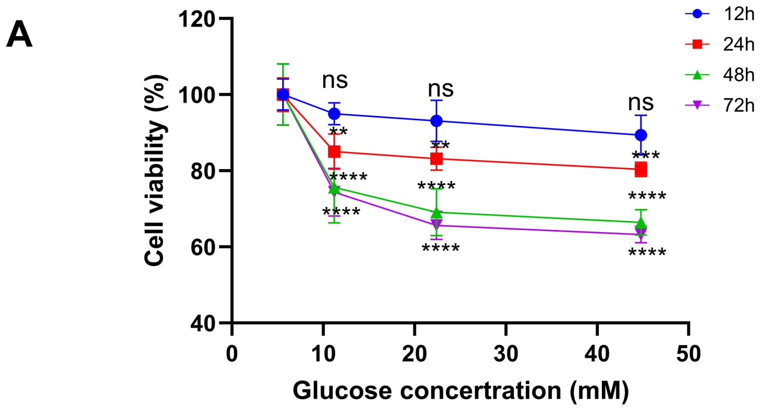

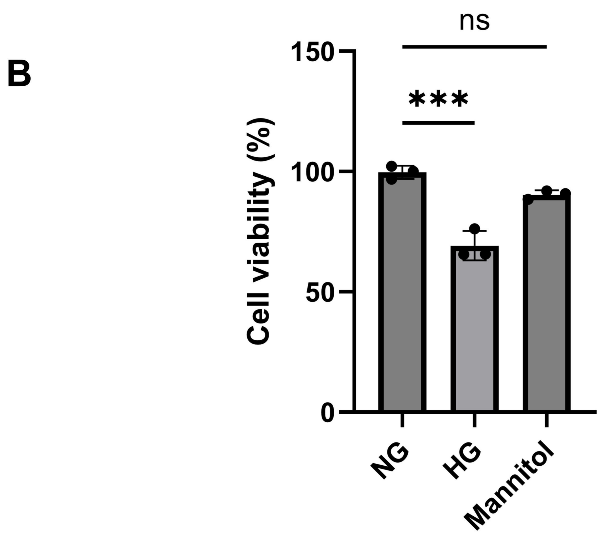

Supplementary Figure 4. Cell viability and osmotic control assessment under high-glucose stress conditions.

(A) CCK-8 assay showing the viability of primary rat chondrocytes exposed to increasing glucose concentrations for 12, 24, 48, or 72 h. Cell viability declined in a concentration- and time-dependent manner. (B) Mannitol osmotic control assessment showing that the observed viability changes under the selected high-glucose stress condition were not attributable to osmotic effects alone. Data are presented as mean ± SD from three independent experiments (n = 3). Statistical significance was evaluated using one-way ANOVA followed by Tukey's post hoc test; *p < 0.05, **p < 0.01, ***p < 0.001, ****p < 0.0001.
